# P311 induces the transdifferentiation of epidermal stem cells to myofibroblast-like cells by stimulating transforming growth factor β1 expression

**DOI:** 10.1186/s13287-016-0421-1

**Published:** 2016-12-01

**Authors:** Haisheng Li, Zhihui Yao, Weifeng He, Hongyan Gao, Yang Bai, Sisi Yang, Lu Zhang, Rixing Zhan, Jianglin Tan, Junyi Zhou, Masao Takata, Jun Wu, Gaoxing Luo

**Affiliations:** 1 Institute of Burn Research, State Key Laboratory of Trauma, Burn and Combined Injury, Southwest Hospital, Third Military Medical University, Chongqing, China; 2 People’s Liberation Army Hospital 59, Kaiyuan, Yunnan Province China; 3 Section of Anaesthetics, Pain Medicine and Intensive Care, Faculty of Medicine, Imperial College London, Chelsea and Westminster Hospital, London, UK

**Keywords:** P311, Epidermal stem cells, Myofibroblast-like cells, Transdifferentiation, Transforming growth factor beta 1

## Abstract

**Background:**

Epithelial to mesenchymal transition, especially to myofibroblasts, plays an important role in wound healing, fibrosis, and carcinogenesis. Epidermal stem cells (EpSCs) are responsible for epidermal renewal and wound re-epithelialization. However, it remains unclear whether and how EpSCs transdifferentiate into myofibroblasts or myofibroblast-like cells (MFLCs). Here, we provide the first evidence showing that P311 induces EpSC to MFLC transdifferentiation (EpMyT) via TGFβ1/Smad signaling.

**Methods:**

Wound healing and mesenchymal features were observed in the P311 KO and P311 WT mouse model of superficial second-degree burns. After the primary human or mouse EpSCs were forced to highly express P311 using an adenoviral vector, EpMyT was observed by immunofluorescence, real-time PCR, and western blot. The activity of TGFβ1 and Smad2/3 in EpSCs with different P311 levels was observed by western blot. The TβRI/II inhibitor LY2109761 and Smad3 siRNA were applied to block the EpMyT in P311-overexpressing EpSCs and exogenous TGFβ1 was to restore the EpMyT in P311 KO EpSCs. Furthermore, the mechanism of P311 regulating TGFβ1 was investigated by bisulfite sequencing PCR, luciferase activity assay, and real-time PCR.

**Results:**

P311 KO mouse wounds showed delayed re-epithelialization and reduced mesenchymal features. The human or mouse EpSCs with overexpressed P311 exhibited fusiform morphological changes, upregulated expression of myofibroblast markers (α-SMA and vimentin), and downregulated expression of EpSC markers (β1-integrin and E-cadherin). P311-expressing EpSCs showed decreased TGFβ1 mRNA and increased TGFβ1 protein, TβRI/II mRNA, and activated Smad2/3. Moreover, LY2109761 and Smad3 siRNA reversed P311-induced EpMyT. Under the stimulation of exogenous TGFβ1, the phosphorylation of Smad2 and Smad3 in P311 KO EpSCs was significantly lower than that in P311 WT EpSCs and the EpMyT in P311 KO EpSCs was restored. Furthermore, P311 enhanced the methylation of TGFβ1 promoter and increased activities of TGFβ1 5′/3′ untranslated regions (UTRs) to stimulate TGFβ1 expression. P311^+^α-SMA^+^ cells and P311^+^vimentin^+^ cells were observed in the epidermis of human burn wounds. Also, P311 was upregulated by IL-1β, IL-6, TNFα, and hypoxia.

**Conclusions:**

P311 is a novel TGFβ1/Smad signaling-mediated regulator of transdifferentiation in EpSCs during cutaneous wound healing. Furthermore, P311 might stimulate TGFβ1 expression by promoting TGFβ1 promoter methylation and by activating the TGFβ1 5′/3′ UTR.

**Electronic supplementary material:**

The online version of this article (doi:10.1186/s13287-016-0421-1) contains supplementary material, which is available to authorized users.

## Background

Cutaneous wounds resulting from injuries such as trauma, burns, surgery, and diabetes are common clinical problems. Inflammatory reactions, granulation tissue formation, and re-epithelialization are the three classic processes during wound healing [1, 2]. Macrophages, neutrophils, and lymphocytes participate in inflammatory responses in wounds. Epidermal stem cells (EpSCs) and keratinocytes perform an essential role in re-epithelialization by migrating from their niche towards the wound and then undergoing proliferation and differentiation [3–5]. Fibroblasts and myofibroblasts have been suggested to be the main functional cells in granulation tissue formation and wound contracture [6]. Myofibroblasts play crucial roles during wound healing, but the healing process can be compromised if the proliferation of myofibroblasts is not properly regulated, which can, in turn, lead to excessive scarring [7]. It is conventionally assumed that myofibroblasts originate from fibroblasts that develop from the mesoderm. However, some studies have shown that epithelial cells from the ectoderm or endoderm can also transdifferentiate into myofibroblasts during pathological processes, for example, during lung, liver, and kidney fibrosis and carcinogenesis [8–10]. Epithelial–mesenchymal transition (EMT) is characterized by the downregulation of epithelial markers (e.g., E-cadherin) and the upregulation of myofibroblast markers (e.g., α-SMA and vimentin). In skin, epidermal cells are classically thought to perform their role in myofibroblast formation by secreting transforming growth factor beta 1 (TGFβ1), interleukin-1 (IL-1), basic fibroblast growth factor (bFGF), tumor necrosis factor alpha (TNFα), and other humoral factors that regulate the fibroblast to myofibroblast differentiation [11–13]. Recently, several studies have shown that epidermal keratinocytes directly transdifferentiate into fibroblasts when exposed to TGFβ1, IL-1β, TNFα, and fetal bovine serum (FBS) [14–16]. However, the mechanisms involved in keratinocyte transdifferentiation during wound healing are not fully understood. Importantly, keratinocytes are the daughter cells of EpSCs that reside in the interfollicular region and EpSCs are primarily responsible for the renewal of the epidermis and wound re-epithelialization [17]. Therefore, it is important to investigate whether and how EpSCs transdifferentiate into myofibroblasts or myofibroblast-like cells (MFLCs).

Using gene expression profiling and comparative proteomic analysis, we found previously that P311 (also known as PTZ17 and neuronal protein 3.1) expression was dramatically increased in hypertrophic scars [18]. The high level of expression of P311 in these tissues suggested that it plays an important role in wound healing and scar formation. P311 is a novel gene that encodes an 8-kDa protein which contains several PEST (rich in proline (P), glutamic acid (D), aspartic acid (E), and serine (S) or threonine (T))-like domains and participates in the development of neurons and smooth muscles [19]. Because myofibroblasts are defined as specialized fibroblasts with smooth muscle-like features, we hypothesized that P311 might also be involved in myofibroblast formation. In fact, our and other studies have shown that P311 can induce primary human fibroblasts [20] and fibroblast cell lines [19, 21] to differentiate into myofibroblasts. We recently found that P311 was highly expressed in EpSCs that are located in the neo-epidermis and hair follicles during wound healing [22] and that P311 promoted EpSC migration. However, the wound healing changes in P311 KO mice and the role of P311 in EpSC to MFLC transdifferentiation (EpMyT) remains unknown.

Previous studies on EMT have shown that TGFβ1 is a factor that induces fibroblasts and epithelial cells to become myofibroblasts. Active TGF-β1 is dissociated from a latent-associated protein (LAP) complex known as LAP-TGFβ1 which is cleaved from a large homodimeric precursor (proTGFβ1). When active TGFβ1 binds its specific receptors, it initiates downstream signaling pathways, such as the Smad pathway [23]. Several studies have shown that P311 is a novel regulator of TGFβ1 in fibroblasts and vascular smooth muscle cells [20, 21, 24]. In this study, we report the first evidence showing that EpSCs can transdifferentiate into MFLCs in vitro and we also show that this process is potentially regulated by P311. Our study provides novel insight into the regulation of transdifferentiation of EpSCs.

## Methods

### Human tissue sources

Paraffin blocks from surgically removed burn wounds and spare normal skin transplants that were obtained from five patients were selected from Department of Pathology archival materials. The samples containing the junctions between normal and burned skin were selected in this study. All of the included five patients were injured by flame or boiling water and the burn sites were the limbs and trunk.

### Animals and superficial second-degree burn mouse model

P311 KO mice were generated and genotyped as described previously [25]. To create a second-degree thermal burn injury, a scalding apparatus was applied using a permanent temperature and pressure (YSL-5Q). A stainless steel cylinder with an area of 2 cm^2^ was heated to 80 °C and placed on the shaved dorsal skin for 3 seconds at a pressure of 500 g [26]. Two wounds were produced on every mouse, and eight mice were included in each group. The wounds were imaged daily using a digital camera and analyzed using ImageJ software. Seven days after injury, the entire wound, including 2 mm of surrounding normal skin, was harvested and separated into two halves across the center. One half of the wound was processed for histological analysis. The other half was rapidly frozen in liquid nitrogen for RNA and protein analysis.

### Culture and characterization of human and mouse EpSCs

Human and mouse EpSCs were isolated from human foreskin and newborn (day 0–2) mouse skin, respectively, as described previously with some modifications [17, 27, 28]. Briefly, the human and mouse skin tissues were incubated in 0.5% Dispase II (04942078001; Roche) in 0.9% NaCl at 4 °C overnight. The epidermal sheet was carefully separated from the dermis, minced, and then digested in 0.5% trypsin in PBS at 37 °C for 10 minutes. The epidermal suspension was gently agitated to create a single-cell suspension. The trypsin was inactivated in calcium-free RPMI 1640 medium containing 10% chelexed FBS. The suspension was filtered through a 70-μm cell strainer (352350; Falcon), and the cell suspension was centrifuged at 800 rpm for 6 minutes. Human cells were resuspended in complete medium containing keratinocyte serum-free medium (K-SFM, 37010-022; Gibco), human recombinant epidermal growth factor (0.18 ng/ml), bovine pituitary extract (28 mg/ml), calcium chloride (0.05 mM), and penicillin and streptomycin solution (100 IU/ml, 15140122; Gibco). The complete medium for mouse EpSCs also contained 10 ng/ml mouse epidermal growth factor (354001; BD) and 10^–10^ M cholera toxin (C9903; Sigma). The cells were seeded at a density of 10^5^ cells/cm^2^ in plates or flasks that were coated with 100 μg/ml type IV collagen (C5533; Sigma) and allowed to adhere for 15 minutes at 37 °C. The nonadherent cells were discarded, and the rapidly adhering cells were cultured in complete medium at 37 °C in 5% CO_2_. The characterization of the EpSCs was performed according to the methods described by Kaur [29]. After the cells were cultured for 4 days, the EpSCs were digested and stained with FITC-conjugated CD71 (561936 and 555536; BD) and PE-conjugated CD49f (555736; BD) antibodies. Flow cytometry data were acquired using an Attune Acoustic Focusing Cytometer (Applied Biosystems, Life Technologies, CA, USA), and the data were analyzed using FlowJo software (Tree Star Incorporation, USA).

### Cell treatments

A replication-defective adenovirus vector encoding human P311 and an empty control vector were prepared as described previously [20]. At approximately 70% cell confluence, 1 × 10^7^ PFU/ml of the viral suspension was added to fresh medium. At 24 hours after transfection, the medium was removed, and the cells were cultured in fresh medium for an additional 48 hours. For the TGFβ1 inhibition experiments, 5 μM LY2109761 (S2704; Selleck) or 100 nM Smad3 siRNA or mock siRNA (siN05815122147; Ribobio) was added to the medium at 24 hours after transfection, and the EpSCs were harvested after 48 hours. The target sequence of Smad3 siRNA was CCGTATGAGCTTCGTCAAA. For the TGFβ1 mRNA stability analysis, 5 μg/ml actinomycinD (1162400; Sigma) was added at 72 hours after transfection, and the cells were subsequently harvested at the indicated time points. For the TGFβ1 restoration and cytokine stimulation experiments, 10 ng/ml exogenous TGFβ1 (PHG9214; Life Technologies), 10 ng/ml IL-1β (AD1413073; R&D systems), 10 ng/ml IL-6 (406-ML-025; R&D systems), or 10 ng/ml TNFα (210-TA-020; R&D systems) was added to EpSCs at 70% cell confluence, and the cells were then cultured for 72 hours. To induce hypoxia, EpSCs at 70% cell confluence were cultured for 72 hours in a mionectic incubator set at 37 °C with 5% CO_2_ and 2% O_2_.

### Immunostaining and morphology

Cultured human and mouse EpSCs were washed in warm PBS and fixed in 4% paraformaldehyde (PFA) for 25 minutes at room temperature. After the cells were washed in PBS, they were blocked in 10% donkey serum and then incubated with primary antibodies at 4 °C overnight. The formalin-fixed and paraffin-embedded human and mouse skins were sectioned and mounted on polylysine-coated slides. The slides were submitted to deparaffinization and rehydration in xylene, ethyl alcohol, and PBS according to a standard protocol. Antigen retrieval was achieved via boiling in 10 mM citrate buffer (pH 6.0) for 15 minutes, followed by blocking and incubating with the antibodies already described. The following primary antibodies were used: CY3-conjugated-α-SMA (1:100, C6198; Sigma), E-cadherin (1:100, SC7870; Santa Cruz), vimentin (1:200, ab92547; Abcam), P311 (1:100, BS0427R; Bioss), and phalloidin (1:1000, 77418-1EA; Sigma). The corresponding secondary antibodies were Alexa Fluor 488 (1:400, A21206; Life Technologies) and Alexa Fluor 594 (1:400, A21207; Life Technologies). Cell nuclei were labeled using DAPI (1:1000, D8417; Sigma), and coverslips were mounted onto the slides using Antifade Mounting Medium (P0126; Beyotime). For immunohistochemistry, the reactions were developed using Biotin-Streptavidin HRP Detection Systems (SP9001; ZSGB-Bio), and color reactions were performed using a diaminobenzidine (DAB) kit (ZLI-9018; ZSGB-Bio). The sections were counterstained with hematoxylin. The negative controls were performed in the same manner but without the primary antibody. For double-staining experiments, immunohistochemistry was first performed for P311 as already described. The tissues were then incubated with the antibodies for vimentin or α-SMA at 37 °C for 1 hour. Subsequent steps were the same as those already described. To quantify wound re-epithelialization, sections from the center of the wounds were stained with HE and then imaged. The epidermal gaps and neo-epidermal lengths were measured using ImageJ 1.48V software (NIH, USA).

### Western blot analysis

Mouse skins were ground in liquid nitrogen and homogenized using a Whole Cell Lysis Kit (KGP2100; Keygen) containing RIPA lysis buffer, protease and phosphatase inhibitor cocktails, and PMSF. Cell lysates were collected at the indicated time points using the same lysis kit. The skin and cell lysates were then centrifuged at 14,000 × *g* for 15 minutes. The supernatants were collected, and protein concentrations were determined using a BCA Assay (23225; Pierce). Equal weights of protein were mixed with SDS loading buffer and cell lysis buffer to obtain equal protein concentrations and then boiled for 8 minutes. Proteins were subjected to electrophoresis in 10% SDS-PAGE gels at 100 V for approximately 2 hours. The separated proteins were transferred electrophoretically to PVDF membranes (Millipore) at 100 V over 90 minutes. The membranes were blocked in 3% bovine serum albumin in Tris-buffered saline containing 0.1% Tween20 (TBST) for 3 hours at room temperature and then incubated with primary antibodies diluted in blocking buffer at 4 °C overnight. The following primary antibodies were used: α-SMA (1:2000, ab5694; Abcam), E-cadherin (1:500, SC7870; Santa Cruz), vimentin (1:2000, ab92547; Abcam), β1-integrin (1:500, SC374429; Santa Cruz), TGFβ1 (1:2000, NB100-91995; Novus), P-Smad2 (1:2000, 3108S; Cell Signaling Technology), Smad2 (1:2000, 5339S; Cell Signaling Technology), P-Smad3 (1:2000, 9520S; Cell Signaling Technology), Smad3 (1:2000, 9523S; Cell Signaling Technology), and GAPDH (1:10,000, KC-5G4; Kangchen). After the membranes were washed in TBST six times for 5 minutes each, they were incubated with horseradish peroxidase-conjugated secondary antibodies at room temperature for 1 hour and then washed again. Finally, the signal was developed using an enhanced chemiluminescence (ECL) detection kit (35055; Pierce) and detected using a Molecular Imager ChemiDoc TMXRS+ Imaging System (BioRad). The protein band densities were analyzed using Image Pro-Plus 6.0 software (Media Cybernetics).

### RNA isolation and real-time PCR

Total RNA was extracted from mouse skin and primary EpSCs with the RNeasy Mini Kit (74104; Qiagen), and cDNA was synthesized with a cDNA Synthesis Kit (FSK-100; Toyobo). Real-time PCR was performed with a SYBR Green Master Mix (QPK-201; Toyobo) on a 7500 Real Time PCR System (Applied Biosystems Instruments). The primers used are presented in Additional file 1: Table S1. The following reaction conditions were used: predenaturation at 95 °C for 2 minutes; and 35 cycles of denaturation at 95 °C for 30 seconds, annealing at 61 °C for 15 seconds, and extension at 72 °C for 32 seconds. GAPDH or HPRT was used as the internal control.

### DNA extraction and bisulfite sequencing PCR

Genomic DNA was extracted from mouse EpSCs using a genomic DNA kit (DP304; TIANGEN). The DNA (1 μg/sample) was converted using an Epitect Bisulfite kit (59104; Qiagen) according to the manufacturer’s instructions. The DNA sequence of the total TGFβ1 promoter (–2000 to +1000) was downloaded from Genebank. The CpG island region and its primer were acquired using Methyl Primer Express (Applied Biosystems Instruments). The bisulfite-converted genomic DNA fragments belonging to the CpG island region (–544 to +990) were then amplified using PCR. The PCR products were resolved on 1% agarose gels, purified, and then cloned with a pMD-19T Vector Cloning Kit (6013; Takara). Five colonies from each ligation were selected randomly and sequenced. The methylation status of each CpG site was analyzed on the QUMA website (http://quma.cdb.riken.jp/).

### Luciferase reporter assay

For the promoter activity assay, the promoter of human TGF-β1 (–1328 to + 812) was cloned into the pGL3-basic plasmid (E1751; Promega). The primers used to clone the TGF-β1 promoter were 5′-GATTCGACGCGTAGATCACTTTGGCTGCTG-3′ and 5′-TAGACCAGATCTGAGCGCGAACAGGGC-3′. For the untranslated region (UTR) binding assay, the 5′ and 3′ UTRs of TGF-β1 were inserted into the firefly luciferase pmirGLO expression vector (E1330; Promega). The primers used to amplify the TGF-β1 UTR were: 5′ UTR, 5′-CTCCCTCCCACCTCCCTCCG-3′ and 5′-GGCCAGGCGTCAGCACCAGTAG-3′; and 3′ UTR, 5′-CCGCTGCCCATCGTGTACTA-3′ and 5′-GGCCTGAACTACTATCTTTTATTGTCTT-3′. Mouse EpSCs were seeded on 12-well plates and transfected with an adenovirus encoding P311. At 24 hours after P311-adenovirus transfection, the cells were transfected with the promoter plasmid or UTR plasmid using Lipofectamine2000 for the promoter or UTR analysis, respectively. After 48 hours, luciferase activity was determined using a Dual Luciferase Assay System (E2940; Promega) and the results were normalized to the internal control Renilla luciferase activity according to the manufacturer’s protocol.

### ELISA

The total amount of TGFβ1 in the cell culture medium was detected by a Quantikine ELISA kit (MB100B; R&D systems) with the EpSC culture supernatant according to the manufacturer’s instructions. Briefly, the culture supernatant was collected by centrifuging the solution at 1000 rpm for 10 minutes. The cell culture supernatant was acidified to obtain total TGFβ1 and then allowed to bind to coated antibodies at room temperature for 2 hours. HRP-conjugated TGFβ1 antibodies were incubated at room temperature for 1.5 hours followed by the addition of chromogen tetramethylbenzidine (TMB) and stop solution. Absorbance was recorded at 450 and 540 nm.

### Statistical analysis

Statistical comparisons were performed using Student's *t* tests or one-way ANOVA by Graphpad Prism 6.0 software (Graphpad Software Inc.). The data are presented as the mean ± standard error of the mean (SEM). In all cases, *P* < 0.05 was considered to indicate statistical significance.

## Results

### Loss of P311 results in altered wound healing kinetics

**Fig. 1:**
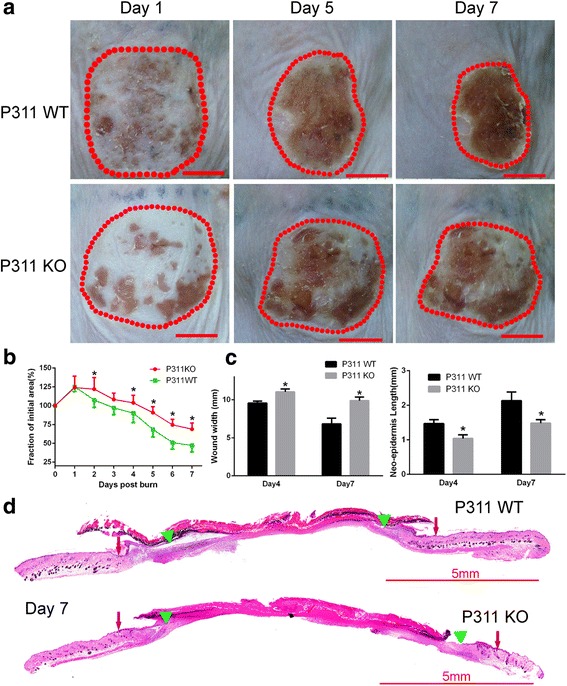
Loss of P311 resulted in altered wound healing kinetics. Superficial second-degree thermal burn wounds were created in the skin of P311 WT and P311 KO mice. **a** Wounds were imaged after the wounding was performed. Representative wounds are shown on days 1, 5, and 7. *Dotted circles* indicate the wound border. **b** Macroscopic quantification of the wound area. Data expressed as a fraction of the initial wound area (day 0). *Error bars* represent SEM, *n* = 8 per group, Student’s *t* test, *P < 0.05. **c** Histological quantification of the wound width (*left*) and the neo-epidermal length (*right*) was performed for the wounds on days 4 and 7. Wound width defined as the distance between one leading edge and another leading edge in the same wound. Neo-epidermal length defined as the distance from the wound margin to the leading edge on the same side of one wound. *P < 0.05 for P311 WT vs P311 KO. **d** Wound re-epithelialization on day 7 using HE staining. Representative images are shown. *Red arrow*, wound margin; *green triangle*, leading edge of the neo-epidermis (Color figure online)

To determine whether P311 could affect burn wound healing, superficial second-degree thermal burn wounds were created in P311 KO mice and sex-matched and age-matched P311 WT mice. On days 5 and 7 post injury, the wound areas in the P311 WT mice were reduced to 69% and 47% of the initial wound area, respectively. However, the average wound area in the P311 KO mice was larger than that in the P311 WT mice (91% and 69% on days 5 and 7, respectively; Fig. 1a, b). HE staining showed that wound re-epithelialization was clearly slower in the P311 KO mice than in the P311 WT mice on days 4 and days 7 post injury (Fig. 1c). The width of each wound in the P311 KO mice was significantly larger than that in the P311 WT mice (P311 KO, 9.87 mm vs P311 WT, 6.82 mm on days 7, *P* < 0.05). Moreover, the average neo-epidermal length in the P311 KO mice was significantly shorter than that in the P311 WT mice (Fig. 1d; P311 KO, 1.48 mm vs P311 WT, 2.14 mm, *P* < 0.05). These results indicate that the deletion of P311 resulted in delayed burn wound healing.

### Mesenchymal features are reduced in P311 KO mouse burn wounds

**Fig. 2:**
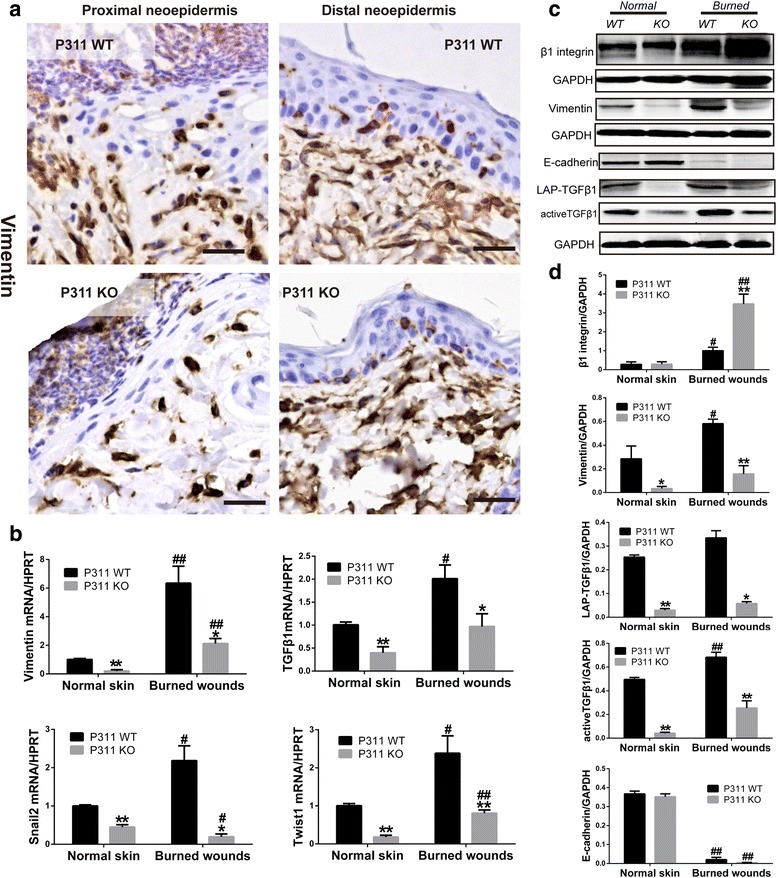
Mesenchymal features in P311 WT and P311 KO mouse burn wounds. **a** Vimentin expression in the proximal and distal neo-epidermis of P311 WT and P311 KO mice was analyzed using immunohistochemistry. Proximal neo-epidermis was defined as the tongue of the neo-epidermis, and distal neo-epidermis was defined as the epidermis at the margin of the wound. *Scale bar*, 25 μm. **b** mRNA levels of vimentin, TGFβ1, Snail2, and Twist1 were detected using real-time PCR in P311 WT and P311 KO mouse burns and normal skin samples. **c** Western blot analysis demonstrating the protein levels of β1-integrin, vimentin, E-cadherin, and active and LAP-bound TGFβ1 in P311 WT and P311 KO mouse burns and normal skin samples. **d** Relative densities of the target proteins were standardized to the expression of GAPDH. Data are shown as the mean ± SEM, Student’s *t* test; *P < 0.05 and **P < 0.01 for P311 WT vs P311 KO; #*P* < 0.05 and ##*P* < 0.01 for normal skin vs burned wounds

Next, we analyzed the mesenchymal features of the cells in mouse burn wounds. IHC staining for vimentin showed that the proximal and distal neo-epidermis in P311 WT mice were both thicker than those in P311 KO mice. Moreover, the expression of vimentin in the proximal and distal neo-epidermis of P311 KO mice was also lower than that in P311 WT mice (Fig. 2a). However, we did not observe specific α-SMA expression in P311 WT or P311 KO mouse wounds (Additional file 2: Figure S1). The results of real-time PCR showed that the mRNA levels of vimentin, the EMT agonist TGFβ1, and the EMT-specific markers Snail2 and Twist1 were significantly higher in normal skin and burn wounds in P311 WT mice than those in P311 KO mice (Fig. 2b), indicating that reprogramming had occurred at the transcriptional level. Western blot analyses showed that the burn wounds contained higher levels of β_1_-integrin than normal skin samples (*P* < 0.05). The expression of β_1_-integrin was significantly higher in burn wounds in the P311 KO mice than that in the P311 WT mice (Fig. 2c, d). The P311 WT burn wounds showed significantly increased levels of vimentin and active TGFβ1 than the normal skin but the P311 KO mice showed no significant difference. The expression levels of vimentin and active and LAP-bound TGFβ1 in P311 KO normal skin or burn wounds were significantly lower than those in P311 WT normal skin or burn wounds (Fig. 2c, d). However, there was no significant difference between E-cadherin levels in P311 KO mice and P311 WT mice, and the E-cadherin levels in normal skin were significantly higher than those in burn wounds (Fig. 2c, d). Taken together, these results hint at a possible relationship between P311 and mesenchymal features in mouse burn wounds.

### P311 induces a myofibroblast-like phenotype in primary mouse and human EpSCs

**Fig. 3:**
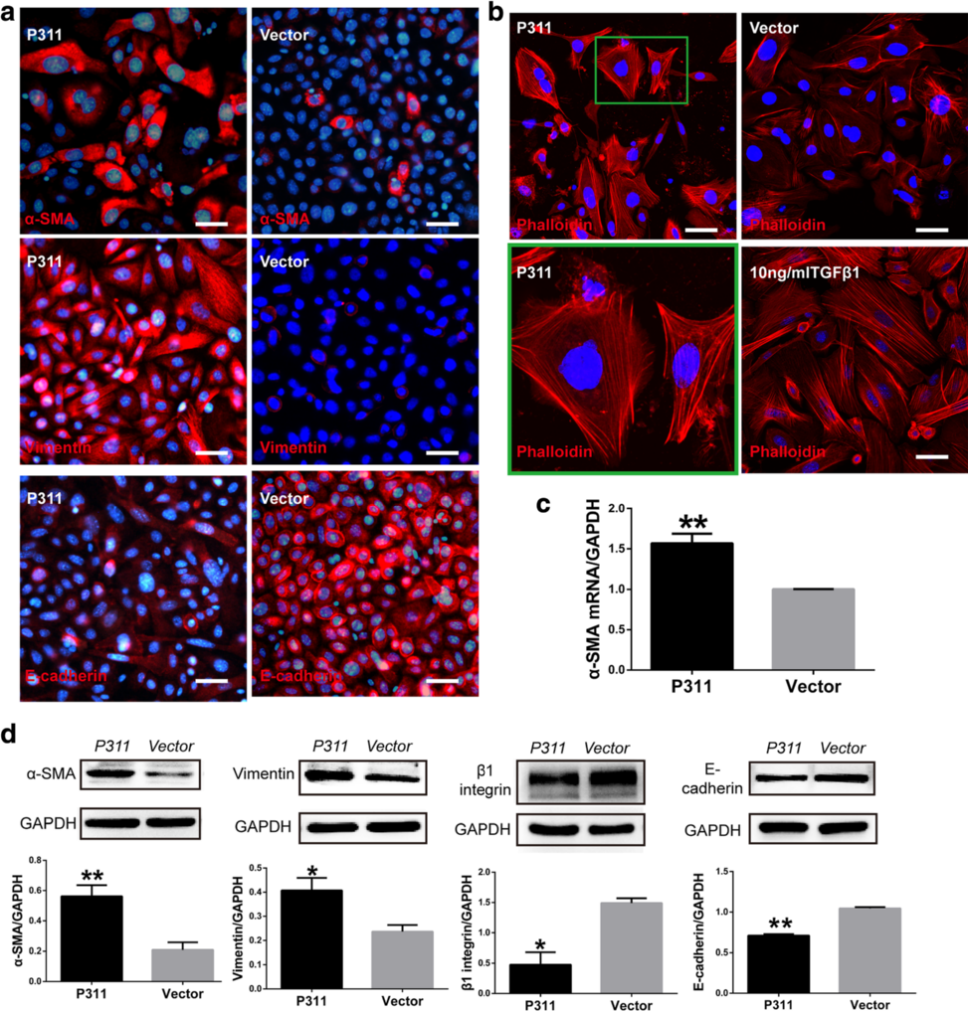
P311 induced a myofibroblast-like phenotype in primary mouse EpSCs. **a** Immunofluorescence for α-SMA (*upper*), vimentin (*middle*), and E-cadherin (*lower*) in EpSCs at 72 hours after P311 transfection (*left*) and control vector transfection (*right*). α-SMA, vimentin, and E-cadherin were labeled using AF594 (*red*), and nuclei were stained with DAPI (*blue*). Representative images from four independent experiments are shown. *Scale bar*, 25 μm. **b** Immunofluorescence for F-actin in EpSCs. TGFβ1 served as the positive control. F-actin was labeled by Rhodamine (*red*), and nuclei were stained with DAPI (*blue*). Details for the observed cell morphologies are shown highlighted in the *interior box* and illustrated in the *lower panel. Scale bar*, 20 μm. **c** The mRNA level of α-SMA was determined in P311-expressing cells and control vector-expressing cells using real-time PCR (*n* = 4). **d** Western blot analysis for α-SMA, vimentin, β1-integerin, and E-cadherin in P311-transfected and control vector-transfected EpSCs. Representative blots from four independent experiments are shown in the *top panel. Bottom panel* shows the quantification of protein levels, which was obtained using densitometry (*n* = 4). Data are represented as mean ± SEM, Student’s *t* test, *P < 0.05 and **P < 0.01 in the P311-transfected group vs the control vector-transfected group (Color figure online)

To determine the effect of P311 on EpMyT, we evaluated primary mouse EpSCs for their EpMyT potential following P311 induction. Mouse EpSCs were isolated from neonatal mice. Cultured cells showed a typical cobblestone-like morphology and were identified as EpSCs using flow cytometry. More than 95% of the cultured cells were CD71^low^CD49f^high^ EpSCs (Additional file 2: Figure S2). The isolated and cultured EpSCs were transfected with an adenoviral expression vector that encoded P311 and GFP. The transfection efficiency of this vector was confirmed by flow cytometry and immunofluorescence (Additional file 2: Figure S3). After 72 hours, the EpSCs began to show pronounced morphological changes. These changes included loosened cell–cell contacts, increased cell volumes, and a fusiform and well-spread morphology that resembled myofibroblasts (Additional file 2: Figure S3A). The control EpSCs continued to display a typical cobblestone appearance. To confirm that P311 induced EpMyT, immunofluorescence, real-time PCR, and western blot assays were performed to detect EpMyT-related markers. After P311 induction, α-SMA was observed in the cytoplasm of the EpSCs, especially in elongated cells (Fig. 3a). Most of the EpSCs expressed vimentin in the perinuclear region (Fig. 3a). Moreover, E-cadherin levels were clearly decreased (Fig. 3a). P311 also induced a dramatic reorganization of the F-actin cytoskeleton, which showed stress fibers and a lamellipodia-like organization. However, the control group EpSCs showed a typical cortical organization that was typical of epidermal cells (Fig. 3b). Real-time PCR showed that the P311-transfected EpSCs contained significantly more α-SMA mRNA than the vector-transfected EpSCs (Fig. 3c; *P* < 0.05). Consistent with these results, the α-SMA and vimentin protein levels in the P311-expressing EpSCs were 2.69-fold and 1.72-fold higher, respectively, than their levels in the control vector-transfected cells (*P* < 0.05). The protein levels of E-cadherin and β1-integrin protein levels were 32% and 68% lower, respectively, than their levels in the control vector-transfected cells (*P* < 0.05; Fig. 3d). We determined that these effects were ubiquitous in human EpSCs (Additional file 2: Figure S4). We also found that P311 promoted EpSC migration (Additional file 2: Figure S5A, B) and induced collagen I and MMP2 expression (Additional file 2: Figure S5C) but had no significant effect on contraction (Additional file 2: Figure S5D). Together, these results show that P311 can induce mouse and human EpSCs to undergo transdifferentiation into MFLCs, not the typical myofibroblasts.

### P311-induced EpMyT is mediated by TGFβ1/Smad signaling

**Fig. 4:**
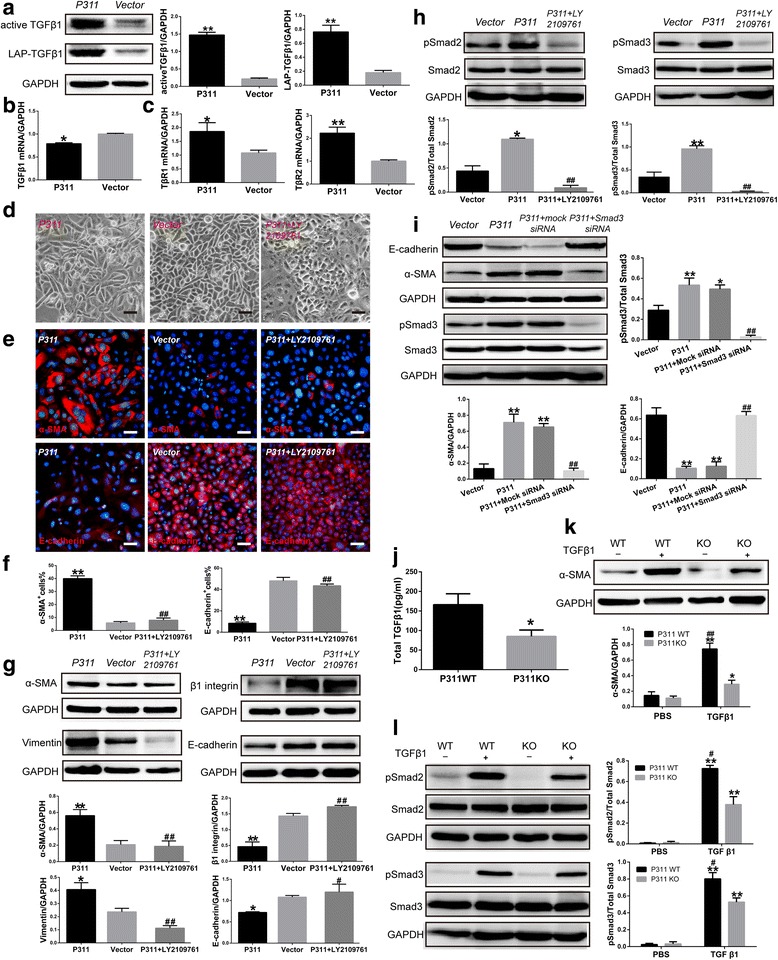
P311-induced EpMyT is mediated by TGFβ1/Smad signaling. **a** Western blot analysis showing expression of LAP-TGFβ1 and active TGFβ1 in primary mouse EpSCs. Relative densities of target proteins are also shown in comparison with the density of GAPDH. **P < 0.01. **b** Real-time PCR showing TGFβ1 mRNA levels in P311-transfected and control vector-transfected EpSCs. *P < 0.05. **c** Real-time PCR showing TβRI and TβRII mRNA levels in P311-transfected and control vector-transfected EpSCs. *P < 0.05 and **P < 0.01. **d** Phase-contrast microscopic observations of cell morphology 72 hours after the EpSCs were transfected with P311-containing vectors either with or without 5 μM LY2109761. **e** Immunofluorescence was performed to determine the expression of α-SMA (*upper*) and E-cadherin (*lower*) in EpSCs at 72 hours after transfection with P311 (*left*), the control vector (*middle*), and P311 with LY2109761 (*right*). α-SMA and E-cadherin were labeled using AF594 (*red*), and nuclei were stained using DAPI (*blue*). *Scale bar*, 25 μm. **f** Quantification of α-SMA^+^ and E-cadherin^+^ cells in an average of five regions. **P < 0.01 for P311 vs control vector; ##*P* < 0.01 for P311 vs P311 + LY2109761. **g** Western blot analysis was performed to determine α-SMA, vimentin, β_1_-integrin, and E-cadherin levels. *P < 0.05 and **P < 0.01 for P311 vs control vector; and #*P* < 0.05 and ##*P* < 0.01 for P311 vs P311 + LY2109761. **h** Western blot analysis showing total and phosphorylated levels of Smad2 and Smad3 in primary EpSCs. LY2109761 was observed to have an inhibitory effect on Smad signals. *P < 0.05 and **P < 0.01 for P311 vs control vector; and ##*P* < 0.01 for P311 vs P311 + LY2109761. **i** Western blot analysis was performed to determine α-SMA, E-cadherin, and total and phosphorylated Smad3 levels after stimulation of Smad3 siRNA.*P < 0.05 and **P < 0.01 for P311 or P311 + mock siRNA vs the control vector; and ##*P* < 0.01 for P311 + Smad3 siRNA vs P311 or P311 + mock siRNA. **j** ELISA showing the level of total TGFβ1 in the culture supernatants of P311-transfected and control vector-transfected EpSCs. *P < 0.05. **k** Western blot analysis showing the expression of α-SMA in primary P311 WT and P311 KO EpSCs after the cells were incubated with or without TGFβ1 treatment. *P < 0.05 and **P < 0.01 relative to PBS control; ##*P* < 0.01 relative to P311 KO cells treated with TGFβ1. **l** Western blot analysis was performed to determine the total and phosphorylated Smad3 levels in P311 KO and P311 WT EpSCs with or without exogenous TGFβ1. Data shown as the mean ± SEM. **P < 0.01 relative to PBS control; #*P* < 0.05 relative to P311 KO cells treated with TGFβ1 (Color figure online)

Because TGFβ1/Smad signaling has ubiquitous effects on cell biology and wound healing, we sought to determine whether P311-induced EpMyT was mediated by TGFβ1/Smad signaling in primary mouse EpSCs. First, the effect of P311 on TGFβ1 expression was investigated using western blot analysis and real-time PCR. The results showed that in P311-expressing EpSCs, the levels of the LAP-bound and active forms of the TGFβ1 protein were significantly higher by 4.21-fold and 6.89-fold, respectively, than the levels in the control vector-transfected cells (Fig. 4a). The level of TGFβ1 mRNA was decreased in P311-expressing EpSCs (Fig. 4b). Meanwhile, in the P311-expressing EpSCs, the mRNA levels of TβRI and TβRII were also upregulated by 1.72-fold and 2.22-fold, respectively, compared with their levels in the control vector-transfected cells (Fig. 4c). Second, to evaluate the contribution of TGFβ1 to P311-induced EpMyT, we introduced LY2109761, a specific small molecule inhibitor of TβRI/II kinase [30]. After LY2109761 was added for 72 hours, the P311-expressing EpSCs lost their myofibroblast morphology and became similar to the control vector-transfected cells (Fig. 4d). Immunofluorescence showed that LY2109761 decreased the number of α-SMA^+^ EpSCs and increased the number of E-cadherin^+^ EpSCs in the P311-transfected cells (Fig. 4e). Quantitative analysis showed that the percentage of α-SMA^+^ cells in the P311-expressing EpSCs was significantly decreased from 40% to 8% in the cells grown in the presence of LY2109761, whereas the percentage of E-cadherin^+^ cells significantly increased from 8% to 43% when cells were incubated in the presence of LY2109761 (Fig. 4f). Consistent with these results, the increases in the levels of the α-SMA and vimentin proteins in the P311-expressing EpSCs were significantly inhibited by LY2109761, whereas the decrease in the levels of the E-cadherin and β_1_-integrin proteins was significantly restored by LY2109761 (Fig. 4g). Also, the increased expression of collagen I and MMP2 in P311-expressing EpSCs were inhibited by LY2109761 (Additional file 2: Figure S5C). Third, the levels of phosphorylated Smad2 and Smad3 were significantly increased in P311-expressing EpSCs. However, the phosphorylation of Smad2 and Smad3 was almost completely inhibited by LY2109761 and total Smad2 and Smad3 were not obviously changed (Fig. 4h and Additional file 2: Figure S6A). Fourth, the specific Smad3 siRNA blocked the P311-induced EpMyT. The inhibitory effect of Smad3 siRNA was confirmed by western blot, which showed that the Smad3 protein level was decreased by over 50% and the phosphorylation of Smad3 was nearly completely inhibited (Fig. 4i and Additional file 2: Figure S6B). Compared with the P311-overexpressed EpSCs and mock siRNA group, the P311-overexpressed EpSCs with Smad3 siRNA exhibited significantly decreased α-SMA and increased E-cadherin (Fig. 4i). Fifth, the addition of exogenous TGFβ1 restored EpMyT in P311 KO EpSCs. The total concentration of TGFβ1 in the culture medium of P311 KO EpSCs was detected using ELISA and found to be significantly lower than the concentration in P311 WT EpSCs (Fig. 4j). The level of the α-SMA protein was lower in the P311 KO EpSCs than in the P311 WT EpSCs but the difference was not significant. In rescue experiments, we found that the level of α-SMA was significantly increased in both P311 KO EpSCs and P311 WT EpSCs (*P* < 0.05, compared with the PBS group) when 10 ng/ml TGFβ1 was added to the medium for 72 hours. Although P311 WT EpSCs continued to express more α-SMA than P311 KO EpSCs following TGFβ1 stimulation (*P* < 0.05), the P311 KO EpSCs that were stimulated with TGFβ1 contained almost the same level of α-SMA protein as the P311 WT EpSCs that were not stimulated with TGFβ1 (*P* > 0.05; Fig. 4k). Sixth, the phosphorylation of Smad2 and Smad3 in P311 KO EpSCs was significantly lower than that in P311 WT EpSCs with the stimulation of exogenous TGFβ1 (Fig. 4l). However, the difference of total Smad2 and Smad3 between P311 WT and P311 KO EpSCs was not significant (Additional file 2: Figure S6C). In conclusion, these results indicate that TGFβ1/Smad signaling is a downstream mediator of P311-induced EpMyT.

### P311 may stimulate TGFβ1 expression by promoting its promoter methylation and UTR activity

**Fig. 5:**
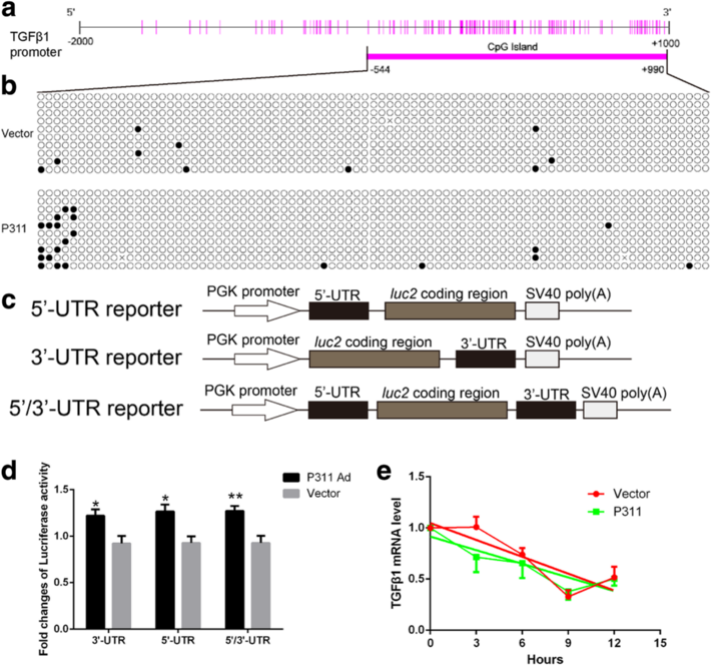
P311 promoted TGFβ1 promoter methylation and UTR activity. **a** Diagram of the CpG island in the mouse TGFβ1 proximal promoter ranging from –544 to +990 relative to the transcription start site. *Vertical bars*, CpG site; *thick horizontal bar*, CpG island. **b** DNA methylation status of the CpG island was analyzed in P311-transfected and control vector-transfected mouse EpSCs using bisulfite sequencing PCR. *Solid circles*, methylated cytosine residues; *empty circles*, unmethylated cytosine residues. **c** pmirGL0 UTR luciferase reporter constructs. **d** Luciferase reporter analysis of the TGFβ1 5′ and 3′ UTRs in mouse EpSCs that were transfected with a P311 adenovirus or an empty control vector (*n* = 3 per group). **e** TGFβ1 mRNA degradation curves. Actinomycin D was added to mouse EpSCs that were transfected with P311 or an empty control vector. The amount of TGFβ1 mRNA was normalized to the initial value at 0 hours (*n* = 4 per group). Data shown as mean ± SEM, Student *t* test, *P < 0.05,**P<0.01. *UTR* untranslated region

To determine the mechanism by which P311 reduced TGFβ1 mRNA levels but elevated TGFβ1 protein levels, the effects of P311 on TGFβ1 transcription (including promoter activity, DNA methylation, and mRNA stability) and translation were analyzed. A luciferase reporter analysis showed that P311 had no effect on TGFβ1 promoter activity (Additional file 2: Figure S7). A DNA sequence analysis revealed that there is a CpG island in the proximal promoter region of *TGFβ1* ranging from –544 to +990 relative to its transcription start site (Fig. 5a). Bisulfite sequencing results showed that the number of methylated CpG sites in the P311-expressing EpSCs was higher than the number in the control vector-transfected cells (Fig. 5b). The effect of P311 on TGFβ1 translation was detected using a luciferase UTR expression system that included the TGFβ1 5′ and 3′ UTRs (Fig. 5c). The results showed that EpSCs which expressed P311 at high levels exhibited higher levels of luciferase activity than were exhibited by the control vector-transfected cells in all of the luciferase UTR expression systems (Fig. 5d). After actinomycin D was added, no significant difference was found between the TGFβ1 mRNA degradation in P311-expressing EpSCs and in the control vector-transfected cells (slope: vector, –0.112 vs P311, –0.0805, *P* = 0.4531; Fig. 5e). Hence, P311 may stimulate TGFβ1 expression by promoting its promoter methylation and UTR activity.

### Mesenchymal features in the epidermis might be associated with P311 activity in human burn wounds

**Fig. 6:**
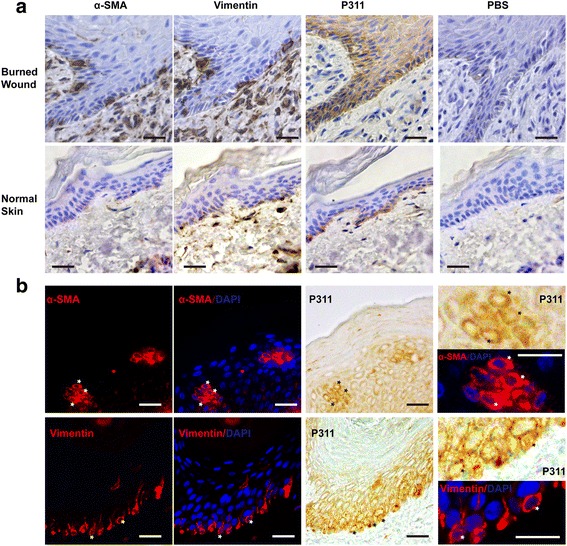
Expression patterns of vimentin, α-SMA, and P311 in the epidermis of human burn wounds. **a** Immunohistochemical staining for vimentin, α-SMA, and P311 was performed using serial sections of human burn wounds and normal skin. For the negative control, the primary antibodies were replaced with PBS. *Scale bar*, 25 μm. **b** Double-labeling for P311 with vimentin or α-SMA was performed using human burn wound tissues. *Double-labeled cells in the epidermis. Cells are shown magnified in the *right panel. Scale bar*, 25 μm

To further explore whether there is a relationship between P311 and EMT in vivo, we detected P311 levels and analyzed mesenchymal features in human burn wounds. First, we performed IHC for P311, vimentin, and α-SMA on serial sections of human burn wounds and normal skin samples. The results showed that while many vimentin^+^ and α-SMA^+^ cells were observed in the epidermis of burn wounds, few such cells were observed in the normal epidermis. Moreover, P311 was broadly expressed in the epidermis of burn wounds, in which it was expressed near to vimentin-expressing and α-SMA-expressing cells (Fig. 6a). Second, double staining for P311 plus vimentin or α-SMA was performed to determine whether P311 is expressed in transformed cells. P311^+^α-SMA^+^ cells and P311^+^vimentin^+^ cells were observed in the epidermis of human burn wounds (Fig. 6b), suggesting that P311 is expressed in α-SMA^+^ and vimentin^+^ epidermal cells. These results thus suggest that mesenchymal features in the epidermis might be associated with P311 activity in human burn wounds.

### P311 is upregulated by several common injury signals

**Fig. 7:**
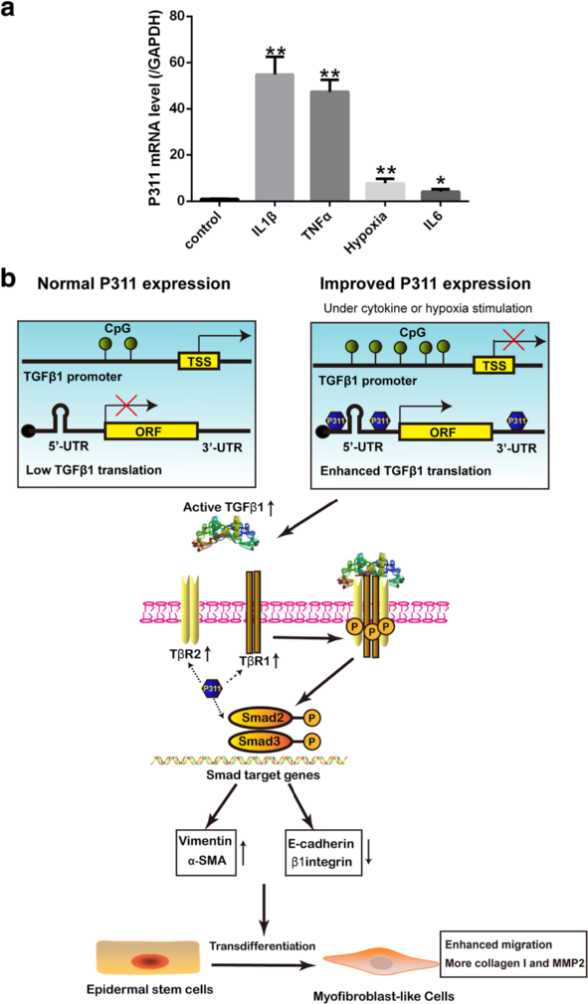
P311 was upregulated by several common injury signals. **a** Primary mouse EpSCs were stimulated using 10 ng/ml IL-1β, IL-6, or TNFα or cultured in 2% O_2_ for 72 hours. Total RNA was extracted from the cells and analyzed using real-time PCR (*n* = 4 per group). Data shown as mean ± SEM, Student’s *t* test; *P < 0.05 and **P < 0.01 vs control group. **b** Model illustrating that P311 is involved in regulating the transdifferentiation of EpSCs into MFLCs via a TGFβ1-dependent process. In a normal state, TGFβ1 transcription is steady but TGFβ1 mRNA is poorly translated because it has long 5′/3′ UTRs. When EpSCs are stimulated with cytokines or hypoxia, P311 is upregulated. Although P311 slightly decreases TGFβ1 transcription by enhancing the methylation of the TGFβ1 promoter, P311 significantly increases the expression of the TGFβ1 protein by activating its 5′/3′ UTRs. Expression of TβRI/II and activity of Smad2/3 are subsequently enhanced indirectly by higher levels of active TGFβ1 or directly by P311. Smad2/3 is then translocated into the nucleus, where it upregulates myofibroblast markers (e.g., vimentin and α-SMA) and downregulates EpSC markers (e.g., E-cadherin and β1integrin) to promote the transdifferentiation of EpSCs into MFLCs. *UTR* untranslated region

To identify which factors promote P311 expression in burn wounds, we analyzed the effects of several common injury signals, including IL-1β, IL-6, TNFα, and hypoxia, on P311 in mouse EpSCs. The P311 mRNA level was significantly increased by the stimulation of IL-1β, IL-6, TNFα, and hypoxia (Fig. 7a). Among the tested factors, IL-1β and TNFα increased P311 expression by more than 40-fold, which was the most effective level of initiation. Hypoxia and IL-6 increased the expression of P311 by 7.58-fold and 4.05-fold, respectively. These results suggest that cytokines and a hypoxic microenvironment might be initiators of P311 expression.

## Discussion

Transdifferentiation of epithelial cells into mesenchymal cells is essential to the development of fibrosis and cancer progression. In the kidneys, EMT is a classic source of myofibroblasts and fibroblasts during renal fibrogenesis. However, little is known regarding the roles performed by and regulation of EpMyT during skin wound healing. Recent studies using rat kidney epithelial cell lines have shown that P311 may promote IL-1α-induced EMT via a TGFβ1-independent pathway [31] and that it may inhibit TGFβ1-induced EMT by blocking the Smad/ILK pathways [32]. Furthermore, our recent in-vivo study showed that P311 promotes renal fibrosis via the TGFβ1/Smad signaling [33]. These controversial results revealed that the roles played by and mechanisms involving P311 in cell biology might be cell and microenvironment specific. However, it remains unclear whether P311 directly induces EpSCs to transdifferentiate into MFLCs. To our knowledge, this is the first study to link P311 with the differentiation potential of stem cells, in which it increases the transdifferentiation of EpSCs into MFLCs. Accordingly, we found that P311 induced EpSCs to transdifferentiate into MFLCs via a TGFβ1-mediated process in vitro. Furthermore, our study also suggests that P311 might regulate TGFβ1 expression by promoting TGFβ1 promoter methylation at the transcriptional level and by activating the TGFβ1 5′/3′ UTRs at the translational level. Moreover, we provide evidence showing that common injury signals (including cytokines and hypoxia) might also promote the expression of P311. Importantly, the deletion of P311 resulted in delayed burn wound healing. We therefore propose that P311 is a novel regulator of the transdifferentiation of EpSCs during skin wound healing (Fig. 7b).

In skin, the majority of research on EMT has focused on tumor progression and skin fibrosis, while only a few studies have shown that EMT may be involved in wound healing [34]. Although EpSCs act as one of the main functional cells in wound healing, it is unclear whether and how EpSCs transdifferentiate into myofibroblasts or MFLCs. In this study, our results indicated that EpSCs were capable of transdifferentiating into MFLCs in vitro and that this process might be regulated by P311. P311-expressing mouse and human EpSCs lost their typical cobblestone morphology and exhibited a myofibroblast-like fusiform morphology. Moreover, P311 upregulated myofibroblast markers (such as vimentin and α-SMA) and downregulated EpSC markers (such as β1-integrin and E-cadherin). Moreover, P311 reorganized the cortical cytoskeleton into filamentous stress fibers. However, functional tests showed that P311 promoted the EpSC migration and the collagen I and MMP2 expression but did not enable EpSCs to develop strong contractility (Additional file 2: Figure S5). One possible explanation for this result is that the EMT program involving P311 might be limited to the early myogenic program, although P311 could induce other programs, such as de-epithelialization (via the loss of E-cadherin), fibrogenesis (via vimentin and collagen I), possible disruption of the basement membrane (via MMP2), and enhanced cell migration. Another possible explanation is that, as reported previously (Additional file 1: Table S2), EMT-sourced myofibroblasts might become the MFLCs that mainly feature motility and secretory abilities, but that are not the same as the classic myofibroblasts which feature both contractility and secretory abilities. Furthermore, we show here that the physiological significance of EpMyT is to enable EpSCs to secrete collagen I and MMP2 and obtain a higher degree of motility [35], but not to generate contractions. Hence, in this study, we defined the transdifferentiated EpSCs as MFLCs to distinguish from the typical myofibroblasts. More studies are needed to determine the mechanisms underlying this interesting phenomenon.

In this study, we found that P311 may activate and amplify TGFβ1/Smad signaling in EpSCs. Specifically, the phosphorylation of Smad2 and Smad3 was increased in P311-overexpressing EpSCs and decreased in the P311 KO EpSCs. To conclusively confirm the role of TGFβ1/Smad signaling in P311-induced EpMyT, loss-of-function and rescue strategies were used. LY2109761, a specific inhibitor of TβRII and TβRI, and the specific Smad3 siRNA almost completely blocked P311-induced EpMyT. Furthermore, the application of exogenous TGFβ1 restored EpMyT in P311 KO EpSCs. The level of α-SMA expression dropped only slightly in P311 KO cells, and exogenous TGFβ1 had a smaller effect on α-SMA in P311 KO cells, suggesting that other factors (such as Rock pathway) may also contribute to this process. These inhibition and rescue experiments further support our hypothesis that P311 promotes EpMyT partly via a TGFβ1-mediated signaling.

The role of and mechanisms involving P311 in the expression of TGFβ1 might be cell specific. In NIH3T3 fibroblasts, P311 inhibited the production of the TGFβ1 mRNA and protein at the post-translational level. Specifically, P311 bound to LAP, which led to a decrease in TGFβ1 auto-induction [36]. In vascular smooth muscle cells, P311 inhibited the transcription of the TGFβ1 mRNA but promoted the production of the TGFβ1 protein at the translational level. In particular, P311 directly bound to the 5′ UTRs of TGFβ1 and eIF3b to stimulate the translation of TGFβ1 [24, 37]. However, these data do not fully explain the observed decrease in TGFβ1 mRNA levels, and the mechanism by which P311 regulates TGFβ1 in EpSCs remains unclear. Our results show that the decrease in TGFβ1 mRNA levels and the increase in TGFβ1 protein levels observed in P311-overexpressing EpSCs are most likely caused by the inhibition of its transcription, which is the result of enhanced promoter methylation and the improved translation of activated 5′ and 3′ UTRs. The current study therefore indicates that P311 regulates TGFβ1 expression not only at the translational level but also at the transcriptional level. This conclusion is also partly supported by our recent finding that eIF6, a partner of P311, downregulates TGFβ1 mRNA and protein expression levels at the transcriptional level via H2A.Z occupancy and Sp1 recruitment in fibroblasts [38]. However, further studies are needed to determine the mechanism by which P311 enhances the methylation of the TGFβ1 promoter and which specific sites of the TGFβ1 5′/3′ UTRs are bound by P311.

In-vivo experiments provided indirect and weak evidence showing that P311 mediates the induction of EpMyT. P311^+^α-SMA^+^ cells and P311^+^vimentin^+^ cells were observed in the epidermis of human burn wounds. Furthermore, the mesenchymal features of these cells were reduced in the P311 KO mouse burn wounds. In this study, we found that superficial second-degree thermal burn wounds in mice healed within 10 days and α-SMA^+^ cells were not detected in the epidermis of these mice, consistent with the results of previous studies [14, 15]. However, the burn wounds obtained from patients who needed skin graft surgery healed slower than the mouse wounds, and α-SMA^+^ cells were observed in the wounds of these patients. Interestingly, α-SMA has also been detected in the epidermis of the wounds of Buruli ulcer patients [39]. Thus, given the results of this study, we concluded that the severity of the injury and the length of time required for wound healing might be related to α-SMA expression. However, there are natural limitations to the detection methods that were used in this study. For example, histological “snapshots” can potentially show nonspecifically bound antibodies, and using full-thickness skin samples means that the mRNA and protein detection methods utilized do not exclude a contribution of the dermis. As a result, we could not determine whether the α-SMA^+^ cells or vimentin^+^ cells did indeed originate from EpSCs in vivo or whether EpMyT was actually reduced in P311 KO mice. Our results only hint that P311 might have a possible relationship with the mesenchymal features of the epidermis in vivo. However, the in-vitro experiments provide direct evidence showing that P311 induced EpMyT. To strengthen the evidence for EpMyT induction in vivo, new fate-mapping studies are needed, and related research is currently being performed in our program.

Cytokines, growth factors, and EMT-related transcription factors are the main reported regulators of EMT [40]. In this study, the relationship between P311 and other EMT inducers (which are also common injury signals), including IL-1β [15], IL-6 [41], TNFα [15], and hypoxia [42], were also investigated. Our results showed that P311 expression was elevated by the investigated injury and EMT inducers, especially IL-1β and TNFα. These results indicate that P311 might be a common pathway for cytokine-induced EMT, and our study adds novel regulators of P311 to previously reported results in the kidney and neural and smooth muscle cells. However, identifying the mechanisms underlying these processes requires further study.

## Conclusions

In summary, this work provides the first evidence showing that P311 is a novel regulator of the potential for EpSCs to transdifferentiate into MFLCs and that this mechanism is mediated by TGFβ1/Smad signaling. Moreover, we demonstrate that P311 might stimulate TGFβ1 expression by promoting TGFβ1 promoter methylation at the transcriptional level and by activating TGFβ1 5′/3′ UTRs at the translational level. Furthermore, P311 might be induced by cytokines and hypoxia, and deletion of P311 resulted in delayed burn wound healing. Thus, we identify the P311–TGFβ1 axis as a novel regulator for transdifferentiation of EpSCs during cutaneous wound healing.

## Additional files

Additional file 1: Supplementary information containing **Table S1.** presenting primer sequences used for real-time PCR, and **Table S2.** presenting the characterization of epithelial to myofibroblast/ myofibroblast-like cell transdifferentiation in 36 articles. (PDF 487 kb)

Additional file 2: Supplementary information containing Supplementary Materials and Methods presenting methods of Gel contraction assay and in-vitro scratch wound assay, **Figure S1.** showing the α-SMA expression in the epidermis of mouse burn wounds, **Figure S2.** showing characterization of primary mouse and human EpSCs, **Figure S3.** showing successful transfection of the P311 adenovirus into mouse EpSCs, **Figure S4.** showing that P311 induced a myofibroblast-like phenotype in primary human EpSCs, **Figure S5.** showing the effect of P311 on the mesenchymal function of EpSCs, **Figure S6.** showing the quantification of the total Smad2 and Smad3 protein, and **Figure S7.** showing the effect of P311 on TGFβ1 promoter activity. (DOC 14540 kb)

## Abbreviations

EMT: Epithelial–mesenchymal transition
EpMyT: Epidermal stem cell to myofibroblast-like cell transdifferentiation
EpSC: Epidermal stem cell
HE: Hematoxylin–eosin
IHC: Immunohistochemistry
MFLC: Myofibroblast-like cell
TGFβ1: Transforming growth factor beta 1
UTR: Untranslated region
