# Supplementary material for: P311 induces the transdifferentiation of epidermal stem cells to myofibroblast-like cells by stimulating transforming growth factor β1 expression
Source: Stem Cell Res Ther. 2016 Dec 1;7:175. doi: 10.1186/s13287-016-0421-1 (PMC5131552; doi:10.1186/s13287-016-0421-1)
Supplement: Supplementary file 1 — Supplementary information containing Table S1. presenting primer sequences used for real-time PCR, and Table S2. presenting the characterization of epithelial to myofibroblast/ myofibroblast-like cell transdifferentiation in 36 articles. (PDF 487 kb) [file 13287_2016_421_MOESM1_ESM.pdf]

**Table S1 Real-time PCR primers**

| Gene | Forward primer | Reverse primer |
|---|---|---|
| α-SMA | 5'-CGTACAACTGGTATTGTGCTGGAC-3' | 5'-TGATGTCACGGACAATCTCACGCT-3' |
| vimentin | 5'-CGTCCACACGCACCTACAG-3' | 5'-GGGGGATGAGGAATAGAGGCT-3' |
| Snail2 | 5'-TGGTCAAGAAACATTTCAACGCC-3' | 5'-GGTGAGGATCTCTGGTTTTG GTA-3' |
| E-cadherin | 5'-CAGGTCTCCTCATGGCTTTGC-3' | 5'-CTTCCGAAAAGAAGGCT GTCC-3' |
| Twist1 | 5'-GGACAAGCTGAGCAAGATTCA-3' | 5'-CGGAGAAGGCGTAGCTGAG-3' |
| TGFβ1 | 5'-CCGCAACAACGCCATCTATG-3' | 5'-CTCTGCACGGGACAGCAA T-3' |
| TβRI | 5'-TCCCAACTACAGGACCTTTTT CA-3' | 5'-GCAGTGGTAAACCTGATCCAGA-3' |
| TβRII | 5'-ATGGAAGAGTGCAACGATTACAT-3' | 5'-TGGCGCAGTTGT CACTGAAAT-3' |
| P311 | 5'-GAGGCTTCCTAAGGGAAGACTT-3' | 5'-AAGTGGAGGTAACTGATTCTTGG-3' |
| GAPDH | 5'-CGTGCCGCCTGGAGAAAC-3' | 5'-AGTGGGAGTTGCTGTTGAAGTC-3' |
| HPRT | 5'-TCAGTCAACGGGGGACATAAA-3' | 5'-GGGGCTGTACTGCTTAACCAG-3'. |

**TableS2 The characterization of epithelial to myofibroblast/myofibroblast-like cell transdifferentiation in 36 articles**

| Authors, year | Treatment | Epithelial cell type | Mesenchymal cell type | Phenotype | | Function | | |
|---|---|---|---|---|---|---|---|---|
| | | | | Up-regulation | Down-regulation | Migration | Contraction | ECM |
| (Zhang et al., 2015) | IL-1alpha | kidney tubular epithelial cells(NRK52E) | myofibroblasts | elongated and fusiform-shaped cells. α-SMA and TGF-β1 | | - | - | - |
| (Xiao et al., 2015) | miR-424 | lung epithelial cell | myofibroblasts | Increased α-SMA, TGF-β1 | no effects on the epithelial or mesenchymal cell markers | - | - | - |
| (Thakur et al., 2015) | TGF-β1 | kidney tubular epithelial cells | myofibroblasts | Increased α-SMA | E-cadherin | - | - | increased fibronectin |
| (O'Connor et al., 2015) | Matrix Rigidity , TGFβ1 | Normal murine mammary gland (NMuMG) epithelial cells | myofibroblasts | myofibroblast marker expression, cell morphology changes, and cytoskeletal reorganization | - | - | - | - |
| (Liu et al., 2014) | hepatitis B virus | human renal tubular epithelial HK-2 cells | myofibroblasts | morphological changes α-SMA,TGF-β1 | E-cadherin | - | - | - |
| (Lechuga et al., 2014) | gamma-CYA siRNA | Human alveolar epithelial cells | myofibroblasts | α-SMA and other contractile proteins | inhibition of genes responsible for cell proliferation | -- | - | - |
| (O'Connor and Gomez, 2013) | (TGF)-β1 | Mouse mammary gland epithelial cells(NMuMG cells) | myofibroblasts | α-SMA, caldesmon, and tropomyosin | - | - | - | - |
| (Gu et al., 2013) | high glucose Fasudil | human renal tubular epithelial (HK-2) cells | myofibroblasts | morphological changes vimentin and α-SMA, TGF-β1 and CTGF | E-cadherin | - | - | - |

| | | | | | | | | |
|---|---|---|---|---|---|---|---|---|
| (Sun et al., 2012) | EGF | GM16 cells | myofibroblasts | Vimentin, and α-SMA | - | - | - | fibronectin |
| (Zhou et al., 2011) | *MeCP2* TGFβ1 | lens epithelial cell | myofibroblasts | α-SMA | - | - | - | - |
| (Liu et al., 2011b) | (IL-1β) and oncostatin M | human renal proximal tubular epithelial cells | myofibroblasts | α-SMA, | CK18 expression | - | - | collagen type I and FN expression |
| (Liu et al., 2011a) | anaphylatoxin C3a, C5a | human renal tubular epithelial (HK-2) cells | myofibroblasts | α-SMA, a slightly spindle-like shape and loss of microvilli on the cell surface. | E-cadherin | - | - | Increased Col-I, TGF-β1 and CTGF |
| (Charbonney et al., 2011) | Contact injury and TGFb | LLC-PK1 (Cl 4) cells, a porcine proximal tubular epithelial cell line | myofibroblasts | α-SMA, | E-cadherin | - | - | - |
| (Aoyagi-Ikeda et al., 2011) | NICD | rat alveolar epithelial cells (RLE-6TN cells) | myofibroblasts | α-SMA,ZO1,VIMENTIN, | E-cadherin,occludin | Increased | - | Collagen I |
| (Xie et al., 2009) | ginsenoside Rg1, TGFb1 | kidney tubular epithelial cells(NRK52E) | myofibroblasts | α-SMA, | E-cadherin | - | - | Collagen I and fibronectin |
| (Shukla et al., 2009) | Hepatocyte growth factor, TGFb1 | Rat alveolar epithelial cells | myofibroblasts | α-SMA, | E-cadherin | - | - | Collagen I and fibronectin |
| (Li et al., 2009) | Hydraulic pressure | kidney tubular epithelial cells(NRK52E) | myofibroblasts | Cell shape, F actin, α-SMA | - | - | - | - |
| (Kim et | α3β1 | In vivo lung | myofibroblasts | α-SMA | proSPC | - | - | Collagen I |

| | | | | | | | | |
|---|---|---|---|---|---|---|---|---|
| al., 2009) | integrin | | | coexpression of GFP with SMA/vimentin /collagen I in transgenic mice | | | | |
| (Sebe et al., 2008) | TGF b1 | porcine proximal tubular cells (LLC-PK/AT1) | myofibroblasts | α-SMA | - | - | - | - |
| (Fan et al., 2007) | TGFb1,$Ca^{2+}$ | LLC-PK1 (CL4) proximal tubular cells | myofibroblasts | α-SMA | - | - | - | - |
| (Forino et al., 2006) | TGFβ1 | human tubular epithelial cells (HUTECs) | Mesenchymal cells , not myofibroblasts | Cell shape, vimentin, and α-SMA. | Cytokeratin, E-cadherin | - | - | CTGF, cadherin 11, collagen III, fibronectin, tenascin and MMP-2) |
| (Sommer et al., 2005) | TGFβ1 TNFα | Renal tubular epithelial cells | myofi broblast-like cells (MFLC) | Cell shape | - | - | - | - |
| (Barth et al., 2005) | Bleomycin | alveolar epithelial cell lines R3/1 and L2 | myofibroblasts | vimentin, α-SMA and caveolin-3 | E-cadherin, aquaporin-5 and cytokeratin 8) | - | - | collagen I/III, fibronectin, Desmin,MMP -2 |
| (Zhang et al., 2004b) (Zhang et al., 2004a) | TGFβ1 CTGF | human proximal tubular epithelial cell line (HKC | myofibroblasts | α-SMA | collagen IV | - | - | Fibronectin , plasminogen activator inhibitor-1(P AI-1) ,tenasci n-C |

| | | | | | | | | |
|---|---|---|---|---|---|---|---|---|
| (Nightingale et al., 2004) | oncostatin M | human proximal tubular epithelial cells | myofibroblasts | α-SMA | E-cadherin, cytokeratin 19 | - | - | collagen I, and fibronectin EDA |
| (Masszi et al., 2004) | TGFβ1 Cell contact | LLC-PK1 (CL4) proximal tubular cells | myofibroblasts | α-SMA | E-cadherin | - | - | fibronectin |
| (Li et al., 2004) | Advanced glycation end products (AGEs) | (NRK52E) | myofibroblasts | α-SMA | E-cadherin | - | - | collagen I and fibronectin |
| (Kim et al., 2004) | serum | lens epithelial cells | myofibroblasts | Cell shape, α-SMA | crystallin | Increased migration ,decreased adhesion | - | collagen I and fibronectin |
| (Yang and Liu, 2002) | TGFβ1 HGF | Human proximal tubular epithelial HKC cells | myofibroblasts | Cell shape, α-SMA,F-actin, Vimentin | E-cadherin | - | - | fibronectin |
| (Yang and Liu, 2001) | TGFβ1 | Human proximal tubular epithelial HKC cells | myofibroblasts | α-SMA, F-actin | E-cadherin | Enhanced Motility and Invasive Capacity | - | *MMP-2* |
| (Oldfield et al., 2001) | TGFβ1 Advanced glycation end products (AGEs) | NRK-52E | myofibroblasts | α-SMA | E-cadherin | - | - | - |

| | | | | | | | | |
|---|---|---|---|---|---|---|---|---|
| (Morishima et al., 2001) | (TGF)-b1 and thrombospondin (TSP)-1 | Airway epithelial cells | myofibroblasts | α-SMA | - | - | - | procollagen type I and III |
| (Fan et al., 2001) | IL 1 | NRK-52E | myofibroblasts | α-SMA, Morphological changes as a loss of apical-basal polarity and microvilli, cell hypertrophy, and the development of an elongated and invasive appearance. | E-cadherin | - | - | - |
| (Fan et al., 1999) | TGF β1 | NRK-52E | myofibroblasts | α-SMA, Cell shape | E-cadherin, double positive by IHC | - | - | - |
| (Lee and Joo, 1999) | TGF-β1 | lens epithelial cells | Mesenchymal cells | α-SMA | - | - | - | collagen I and fibronectin |

Note: '-' represented not detected.

**References:**


1. Aoyagi-Ikeda, K., Maeno, T., Matsui, H., Ueno, M., Hara, K., Aoki, Y., Aoki, F., Shimizu, T., Doi, H., Kawai-Kowase, K., *et al.* (2011). Notch induces myofibroblast differentiation of alveolar epithelial cells via transforming growth factor-{β}-Smad3 pathway. American journal of respiratory cell and molecular biology *45*, 136-144.
2. Barth, K., Reh, J., Sturrock, A., and Kasper, M. (2005). Epithelial vs myofibroblast differentiation in immortal rat lung cell lines--modulating effects of bleomycin. Histochemistry and cell biology *124*, 453-464.
3. Charbonney, E., Speight, P., Masszi, A., Nakano, H., and Kapus, A. (2011). beta-catenin and Smad3 regulate the activity and stability of myocardin-related transcription factor during epithelial-myofibroblast transition. Molecular biology of the cell *22*, 4472-4485.
4. Fan, J.M., Huang, X.R., Ng, Y.Y., Nikolic-Paterson, D.J., Mu, W., Atkins, R.C., and Lan, H.Y. (2001). Interleukin-1 induces tubular epithelial-myofibroblast transdifferentiation through a transforming growth factor-beta1-dependent mechanism in vitro. American journal of kidney diseases : the official journal of the National Kidney Foundation *37*, 820-831.
5. Fan, J.M., Ng, Y.Y., Hill, P.A., Nikolic-Paterson, D.J., Mu, W., Atkins, R.C., and Lan, H.Y. (1999). Transforming growth factor-beta regulates tubular epithelial-myofibroblast transdifferentiation in vitro. Kidney Int *56*, 1455-1467.
6. Fan, L., Sebe, A., Peterfi, Z., Masszi, A., Thirone, A.C., Rotstein, O.D., Nakano, H., McCulloch, C.A., Szaszi, K., Mucsi, I., *et al.* (2007). Cell contact-dependent regulation of epithelial-myofibroblast transition via the rho-rho kinase-phospho-myosin pathway. Molecular biology of the cell *18*, 1083-1097.
7. Forino, M., Torregrossa, R., Ceol, M., Murer, L., Della Vella, M., Del Prete, D., D'Angelo, A., and Anglani, F. (2006). TGFbeta1 induces epithelial-mesenchymal transition, but not

myofibroblast transdifferentiation of human kidney tubular epithelial cells in primary culture. International journal of experimental pathology *87*, 197-208.

8. Gu, L., Gao, Q., Ni, L., Wang, M., and Shen, F. (2013). Fasudil inhibits epithelial-myofibroblast transdifferentiation of human renal tubular epithelial HK-2 cells induced by high glucose. Chemical & pharmaceutical bulletin *61*, 688-694.
9. Kim, J.T., Lee, E.H., Chung, K.H., Kang, I.C., Lee, D.H., and Joo, C.K. (2004). Transdifferentiation of cultured bovine lens epithelial cells into myofibroblast-like cells by serum modulation. Yonsei medical journal *45*, 380-391.
10. Kim, K.K., Wei, Y., Szekeres, C., Kugler, M.C., Wolters, P.J., Hill, M.L., Frank, J.A., Brumwell, A.N., Wheeler, S.E., Kreidberg, J.A.*, et al.* (2009). Epithelial cell alpha3beta1 integrin links beta-catenin and Smad signaling to promote myofibroblast formation and pulmonary fibrosis. The Journal of clinical investigation *119*, 213-224.
11. Lechuga, S., Baranwal, S., Li, C., Naydenov, N.G., Kuemmerle, J.F., Dugina, V., Chaponnier, C., and Ivanov, A.I. (2014). Loss of gamma-cytoplasmic actin triggers myofibroblast transition of human epithelial cells. Molecular biology of the cell *25*, 3133-3146.
12. Lee, E.H., and Joo, C.K. (1999). Role of transforming growth factor-beta in transdifferentiation and fibrosis of lens epithelial cells. Investigative ophthalmology & visual science *40*, 2025-2032.
13. Li, F.Y., Xie, X.S., Fan, J.M., Li, Z., Wu, J., and Zheng, R. (2009). Hydraulic pressure inducing renal tubular epithelial-myofibroblast transdifferentiation in vitro. Journal of Zhejiang University Science B *10*, 659-667.
14. Li, J.H., Wang, W., Huang, X.R., Oldfield, M., Schmidt, A.M., Cooper, M.E., and Lan, H.Y. (2004). Advanced glycation end products induce tubular epithelial-myofibroblast transition through the RAGE-ERK1/2 MAP kinase signaling pathway. The American journal of pathology *164*, 1389-1397.
15. Liu, C., Chen, F., Han, X., Xu, H., and Wang, Y. (2014). Role of TGF-beta1/p38 MAPK pathway in hepatitis B virus-induced tubular epithelial-myofibroblast transdifferentiation. International journal of clinical and experimental pathology *7*, 7923-7930.
16. Liu, F., Gou, R., Huang, J., Fu, P., Chen, F., Fan, W.X., Huang, Y.Q., Zang, L., Wu, M., Qiu, H.Y.*, et al.* (2011a). Effect of anaphylatoxin C3a, C5a on the tubular epithelial-myofibroblast transdifferentiation in vitro. Chinese medical journal *124*, 4039-4045.
17. Liu, Q., Liu, S., Shi, Y., Li, H., Hao, J., Xing, L., Cao, Y., and Duan, H. (2011b). Suppressors of cytokine signaling inhibit tubular epithelial cell-myofibroblast transdifferentiation. American journal of nephrology *34*, 142-151.
18. Masszi, A., Fan, L., Rosivall, L., McCulloch, C.A., Rotstein, O.D., Mucsi, I., and Kapus, A. (2004). Integrity of cell-cell contacts is a critical regulator of TGF-beta 1-induced epithelial-to-myofibroblast transition: role for beta-catenin. The American journal of pathology *165*, 1955-1967.
19. Morishima, Y., Nomura, A., Uchida, Y., Noguchi, Y., Sakamoto, T., Ishii, Y., Goto, Y., Masuyama, K., Zhang, M.J., Hirano, K.*, et al.* (2001). Triggering the induction of myofibroblast and fibrogenesis by airway epithelial shedding. American journal of respiratory cell and molecular biology *24*, 1-11.
20. Nightingale, J., Patel, S., Suzuki, N., Buxton, R., Takagi, K.I., Suzuki, J., Sumi, Y., Imaizumi, A., Mason, R.M., and Zhang, Z. (2004). Oncostatin M, a cytokine released by activated mononuclear cells, induces epithelial cell-myofibroblast transdifferentiation via Jak/Stat pathway activation. Journal of the American Society of Nephrology : JASN *15*, 21-32.
21. O'Connor, J.W., and Gomez, E.W. (2013). Cell adhesion and shape regulate TGF-beta1-induced epithelial-myofibroblast transition via MRTF-A signaling. PloS one *8*, e83188.
22. O'Connor, J.W., Riley, P.N., Nalluri, S.M., Ashar, P.K., and Gomez, E.W. (2015). Matrix Rigidity Mediates TGFbeta1-Induced Epithelial-Myofibroblast Transition by Controlling Cytoskeletal Organization and MRTF-A Localization. Journal of cellular physiology *230*, 1829-1839.
23. Oldfield, M.D., Bach, L.A., Forbes, J.M., Nikolic-Paterson, D., McRobert, A., Thallas, V., Atkins, R.C., Osicka, T., Jerums, G., and Cooper, M.E. (2001). Advanced glycation end products cause

epithelial-myofibroblast transdifferentiation via the receptor for advanced glycation end products (RAGE). The Journal of clinical investigation *108*, 1853-1863.

24. Sebe, A., Leivonen, S.K., Fintha, A., Masszi, A., Rosivall, L., Kahari, V.M., and Mucsi, I. (2008). Transforming growth factor-beta-induced alpha-smooth muscle cell actin expression in renal proximal tubular cells is regulated by p38beta mitogen-activated protein kinase, extracellular signal-regulated protein kinase1,2 and the Smad signalling during epithelial-myofibroblast transdifferentiation. Nephrology, dialysis, transplantation : official publication of the European Dialysis and Transplant Association - European Renal Association *23*, 1537-1545.
25. Shukla, M.N., Rose, J.L., Ray, R., Lathrop, K.L., Ray, A., and Ray, P. (2009). Hepatocyte growth factor inhibits epithelial to myofibroblast transition in lung cells via Smad7. American journal of respiratory cell and molecular biology *40*, 643-653.
26. Sommer, M., Gerth, J., Stein, G., and Wolf, G. (2005). Transdifferentiation of endothelial and renal tubular epithelial cells into myofibroblast-like cells under in vitro conditions: a morphological analysis. Cells, tissues, organs *180*, 204-214.
27. Sun, Q., Sattayakhom, A., Backs, J., Stremmel, W., and Chamulitrat, W. (2012). Role of myocyte enhancing factor 2B in epithelial myofibroblast transition of human gingival keratinocytes. Experimental biology and medicine (Maywood, NJ) *237*, 178-185.
28. Thakur, S., Viswanadhapalli, S., Kopp, J.B., Shi, Q., Barnes, J.L., Block, K., Gorin, Y., and Abboud, H.E. (2015). Activation of AMP-activated protein kinase prevents TGF-beta1-induced epithelial-mesenchymal transition and myofibroblast activation. The American journal of pathology *185*, 2168-2180.
29. Xiao, X., Huang, C., Zhao, C., Gou, X., Senavirathna, L.K., Hinsdale, M., Lloyd, P., and Liu, L. (2015). Regulation of myofibroblast differentiation by miR-424 during epithelial-to-mesenchymal transition. Archives of biochemistry and biophysics *566*, 49-57.
30. Xie, X.S., Yang, M., Liu, H.C., Zuo, C., Li, H.J., and Fan, J.M. (2009). Ginsenoside Rg1, a major active component isolated from Panax notoginseng, restrains tubular epithelial to myofibroblast transition in vitro. Journal of ethnopharmacology *122*, 35-41.
31. Yang, J., and Liu, Y. (2001). Dissection of key events in tubular epithelial to myofibroblast transition and its implications in renal interstitial fibrosis. The American journal of pathology *159*, 1465-1475.
32. Yang, J., and Liu, Y. (2002). Blockage of tubular epithelial to myofibroblast transition by hepatocyte growth factor prevents renal interstitial fibrosis. Journal of the American Society of Nephrology : JASN *13*, 96-107.
33. Zhang, C., Meng, X., Zhu, Z., Liu, J., and Deng, A. (2004a). Connective tissue growth factor regulates the key events in tubular epithelial to myofibroblast transition in vitro. Cell biology international *28*, 863-873.
34. Zhang, C., Meng, X., Zhu, Z., Yang, X., and Deng, A. (2004b). Role of connective tissue growth factor in renal tubular epithelial-myofibroblast transdifferentiation and extracellular matrix accumulation in vitro. Life Sci *75*, 367-379.
35. Zhang, Y., Ma, X., Xie, X., Sun, G., Liang, W., Li, X., Wang, F., Zhang, L., Yan, B., and Fan, J. (2015). Role of P311 in interleukin-1alpha-induced epithelial to myofibroblast transition in kidney tubular epithelial cells. Renal failure *37*, 1384-1389.
36. Zhou, P., Lu, Y., and Sun, X.H. (2011). Zebularine suppresses TGF-beta-induced lens epithelial cell-myofibroblast transdifferentiation by inhibiting MeCP2. Molecular vision *17*, 2717-2723.
