# Supplementary material for: P311 induces the transdifferentiation of epidermal stem cells to myofibroblast-like cells by stimulating transforming growth factor β1 expression
Source: Stem Cell Res Ther. 2016 Dec 1;7:175. doi: 10.1186/s13287-016-0421-1 (PMC5131552; doi:10.1186/s13287-016-0421-1)
Supplement: Supplementary file 2 — Supplementary information containing Supplementary Materials and Methods presenting methods of Gel contraction assay and in-vitro scratch wound assay, Figure S1. showing the α-SMA expression in the epidermis of mouse burn wounds, Figure S2. showing characterization of primary mouse and human EpSCs, Figure S3. showing successful transfection of the P311 adenovirus into mouse EpSCs, Figure S4. showing that P311 induced a myofibroblast-like phenotype in primary human EpSCs, Figure S5. showing the effect of P311 on the mesenchymal function of EpSCs, Figure S6. showing the quantification of the total Smad2 and Smad3 protein, and Figure S7. showing the effect of P311 on TGFβ1 promoter activity. (DOC 14540 kb) [file 13287_2016_421_MOESM2_ESM.doc]

**P311 induces the transdifferentiation of epidermal stem cells to myofibroblast-like cells by stimulating transforming growth factor β1 expression**

Haisheng Li^1^, Zhihui Yao^1^,Weifeng He^1^, Hongyan Gao^1^, Yang Bai^1^, Sisi Yang^1^, Lu Zhang^1^, Rixing Zhan^1^, Jianglin Tan^1^, Junyi Zhou^1^, Masao Takata^2^, Jun Wu^1^*, Gaoxing Luo^1^*

**Supplementary Information**

**Supplementary Materials and methods**

**Gel contraction assay**

The collagen was extracted from rat tails as previously and the concentration of collagen in 0.5M acetic acid was adjusted to 5mg/ml. The EpSC populated gel contraction analysis was performed as previously with some modifications. Briefly, mouse EpSCs were cultured and transfected with P311-expressing or vector control adenovirus as described above. Then, EpSCs were harvested by trypsin and the cell density was determined. The 0.5mg/ml collagen gel was prepared by mixing 100μL collagen stock solution, 50μL 1M NaOH, 100μL 10×RMPI1640 and 750μL H_2_O on ice. The mixture was added to the 24-well plate and placed in room temperature for 10 minutes for gel formation. Then, 1×10^4^ cells were seeded on the surface of collagen gel and the medium was changed every two days. The collagen gel was photographed every day until one week. The contraction index was calculated by the faction of the gel area at days 7 in the gel area at day 0.

**In vitro scratch wound assay**

Briefly, EpSCs were seeded in 12-well plates and tranfected with virus as previously. Until 90% confluence, cells are treated with 4 ng/ml mitomycin C (Sigma, USA, M4287) for 2 hours to suppress cell proliferation and then the cell monolayer is scratched using a 200μL disposable plastic pipette tip. The cells were observed 0, 24, 48 hours after the scratching, and the cells were photographed using an inverted microscope (OLYMPUS, Japan, IX71).

**References**

1. Wang Y, Xu R, Luo G, Lei Q, Shu Q, Yao Z, Li H, Zhou J, Tan J, Yang S, et al: Biomimetic fibroblast-loaded artificial dermis with "sandwich" structure and designed gradient pore sizes promotes wound healing by favoring granulation tissue formation and wound re-epithelialization. *Acta Biomater* 2016, 30:246-257.

2. Souren JM, Ponec M, van Wijk R: Contraction of collagen by human fibroblasts and keratinocytes. *In Vitro Cell Dev Biol* 1989, 25:1039-1045.

**Supplementary figures and figure legends**


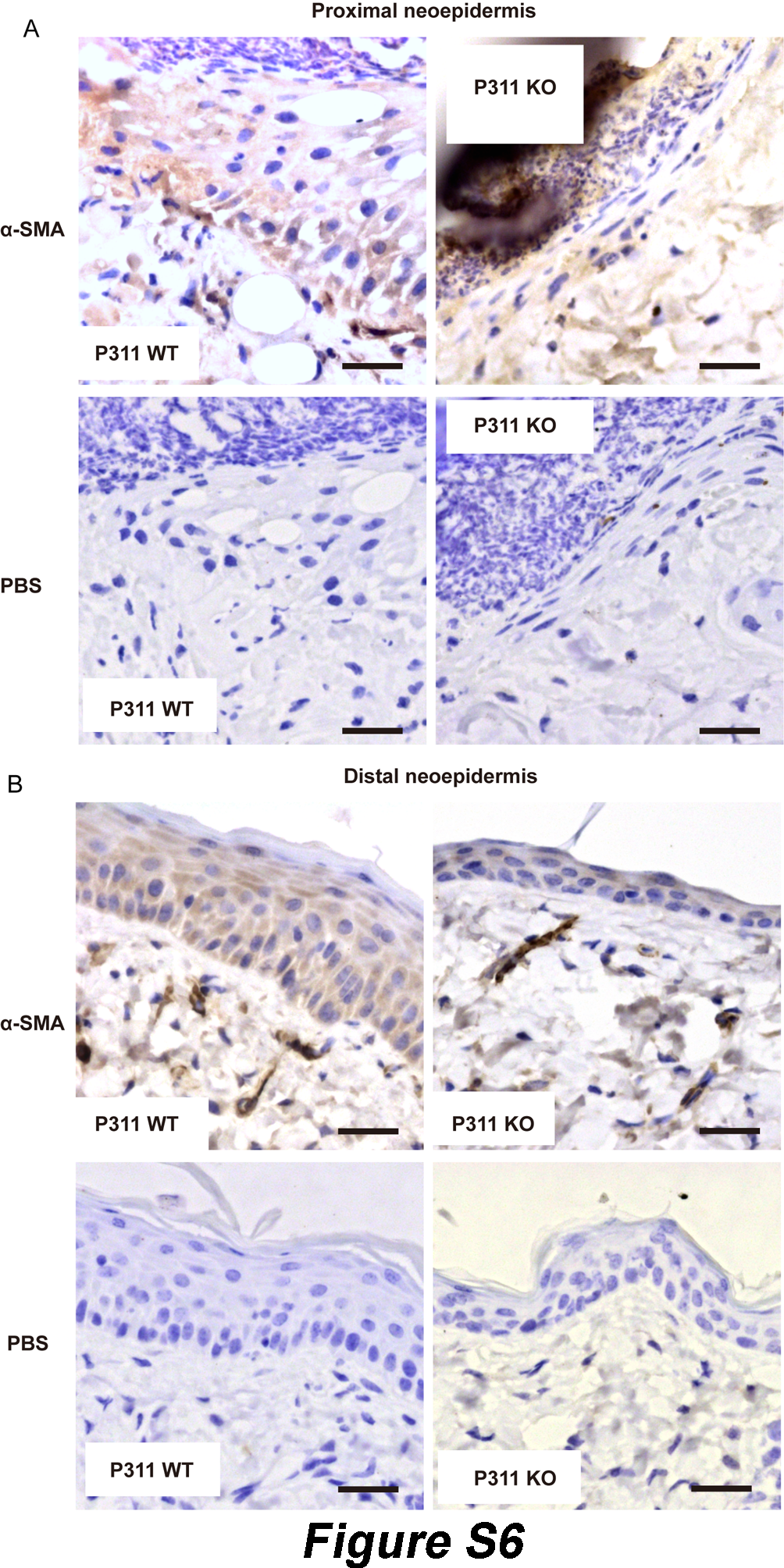


**Figure S1. The α-SMA expression in the epidermis of mouse burn wounds.**

Immunohistochemistry was performed to analyze α-SMA levels in P311 WT and P311 KO mouse wounds at day seven post-burn. **A.** α-SMA expression in the proximal neo-epidermis, which was defined as the tongue of neo-epidermis. **B.** α-SMA expression in the distal neo-epidermis, which was defined as the margin of wound. The primary antibodies were replaced with PBS to perform the negative controls. Scale bar, 25 μm


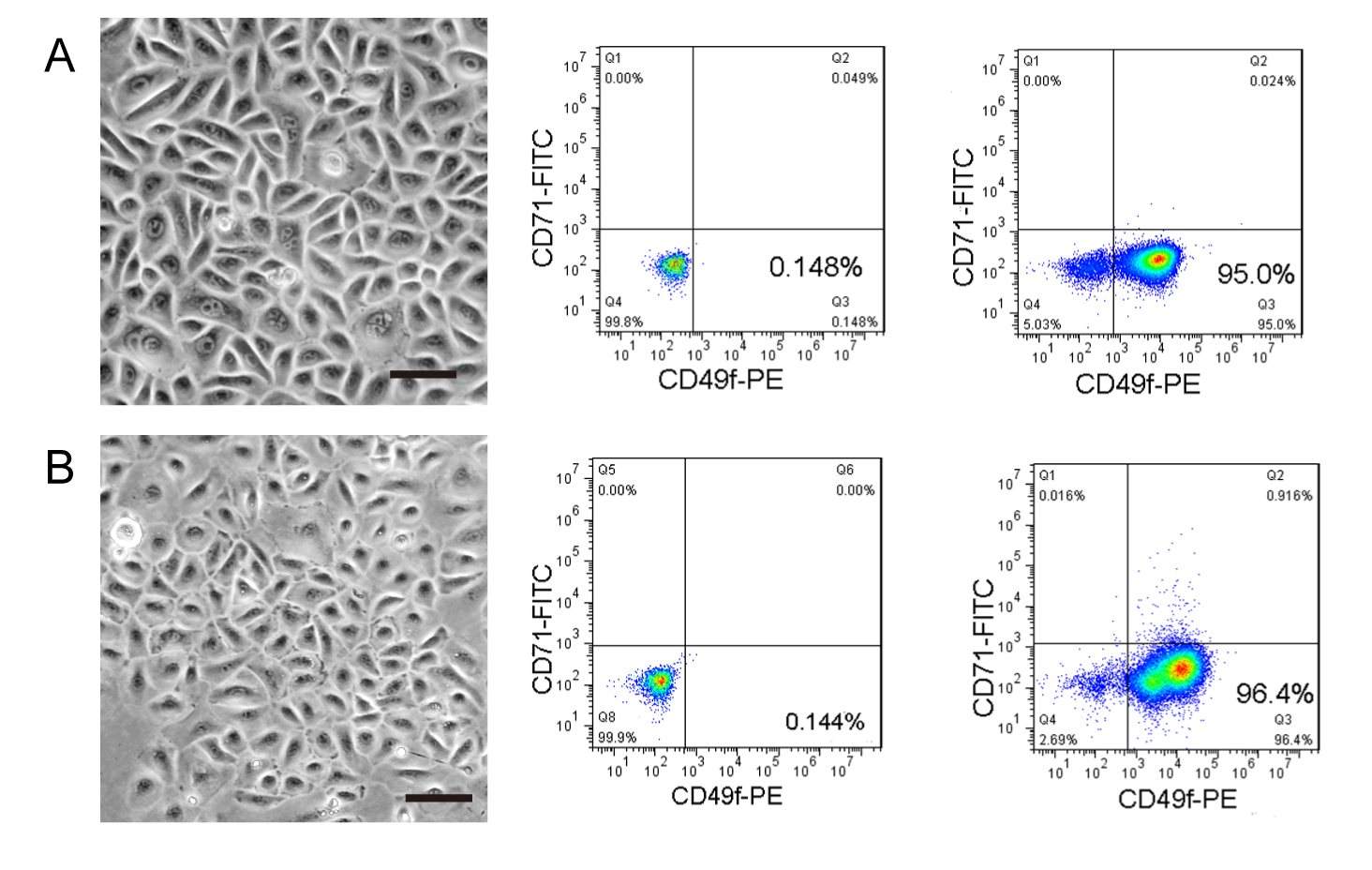


**Figure S2. Characterization of primary mouse and human EpSCs.**

**A.** Human EpSC morphology was analyzed using phase contrast microscopy (left) and flow cytometry in CD71^low^CD49f^high^ EpSCs (right) after 6-7 days in culture. The isotype control is shown in the middle panel. **B.** Mouse EpSC morphology was analyzed using phase contrast microscopy (left) and flow cytometry in CD71^low^CD49f^high^ EpSCs (right) after 4-5 days in culture. The isotype controls are shown in the middle panel. Scale bar, 25 μm.


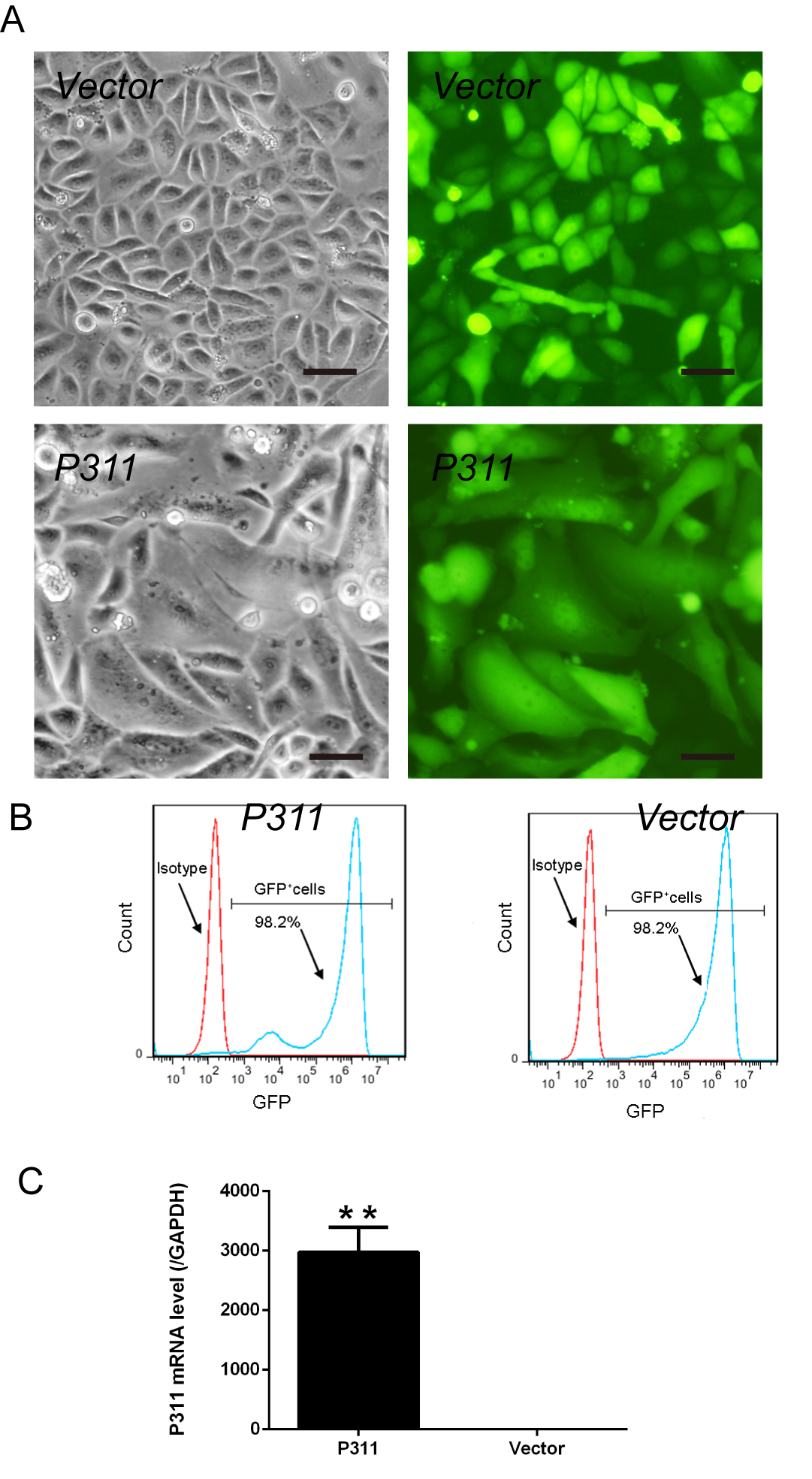


**Figure S3. Successful transfection of the P311 adenovirus into mouse EpSCs**

**A.** Mouse EpSC morphology was analyzed using phase contrast microscopy (left) and fluorescent microscopy (right). Scale bar, 25 μm. **B**. Flow cytometry was used to analyze the GFP^+^ fraction of cells in the P311-transfected EpSCs (left) and the control vector-transfected EpSCs (right) to confirm transfection efficiency.


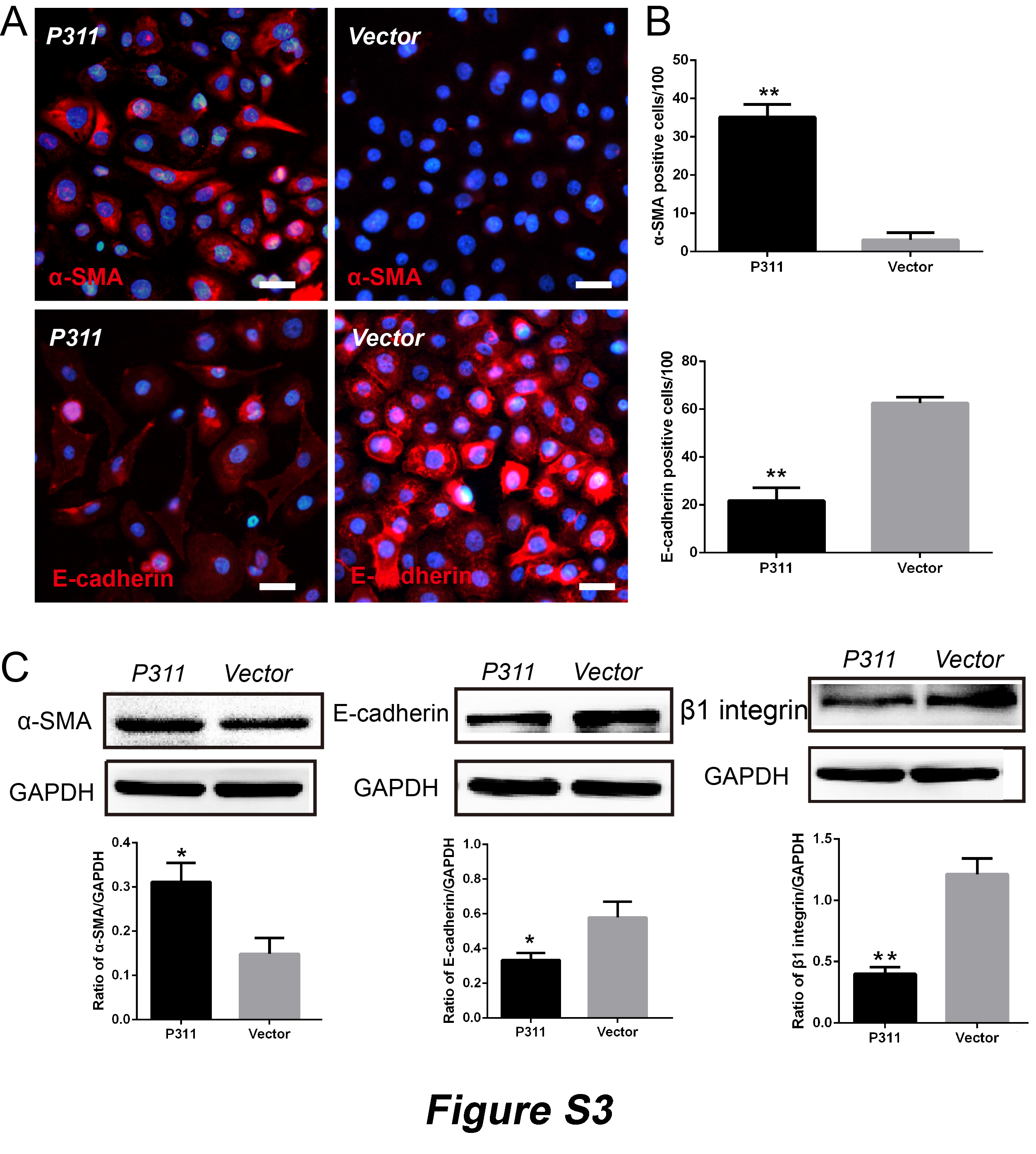


**Figure S4. P311 induced a myofibroblast-like phenotype in primary human EpSCs.**

**A.** Immunofluorescence was performed to analyze the expression of α-SMA (upper) and E-cadherin (lower) in EpSCs at 72 hours after transfection with P311 (left) and control vector adenoviruses (middle). α-SMA/E-cadherin were labeled using AF594 (red), and nuclei were stained using DAPI (blue). Representative images from four independent experiments are shown. Scale bar, 25 μm. **B.** Quantification of α-SMA- and E-cadherin**-**positive cells was performed by averaging the values from five regions. **C.** Western blot analysis was performed to determine α-SMA, β1-integerin and E-cadherin levels in P311-transfected and control vector-transfected EpSCs. The relative densities of the target proteins were compared to that of the control, GAPDH, which is shown in the right panel (n = 4 per group). The data represent the mean ± SEM, Student’s t-test; * P<0.05, ** P<0.01.

**
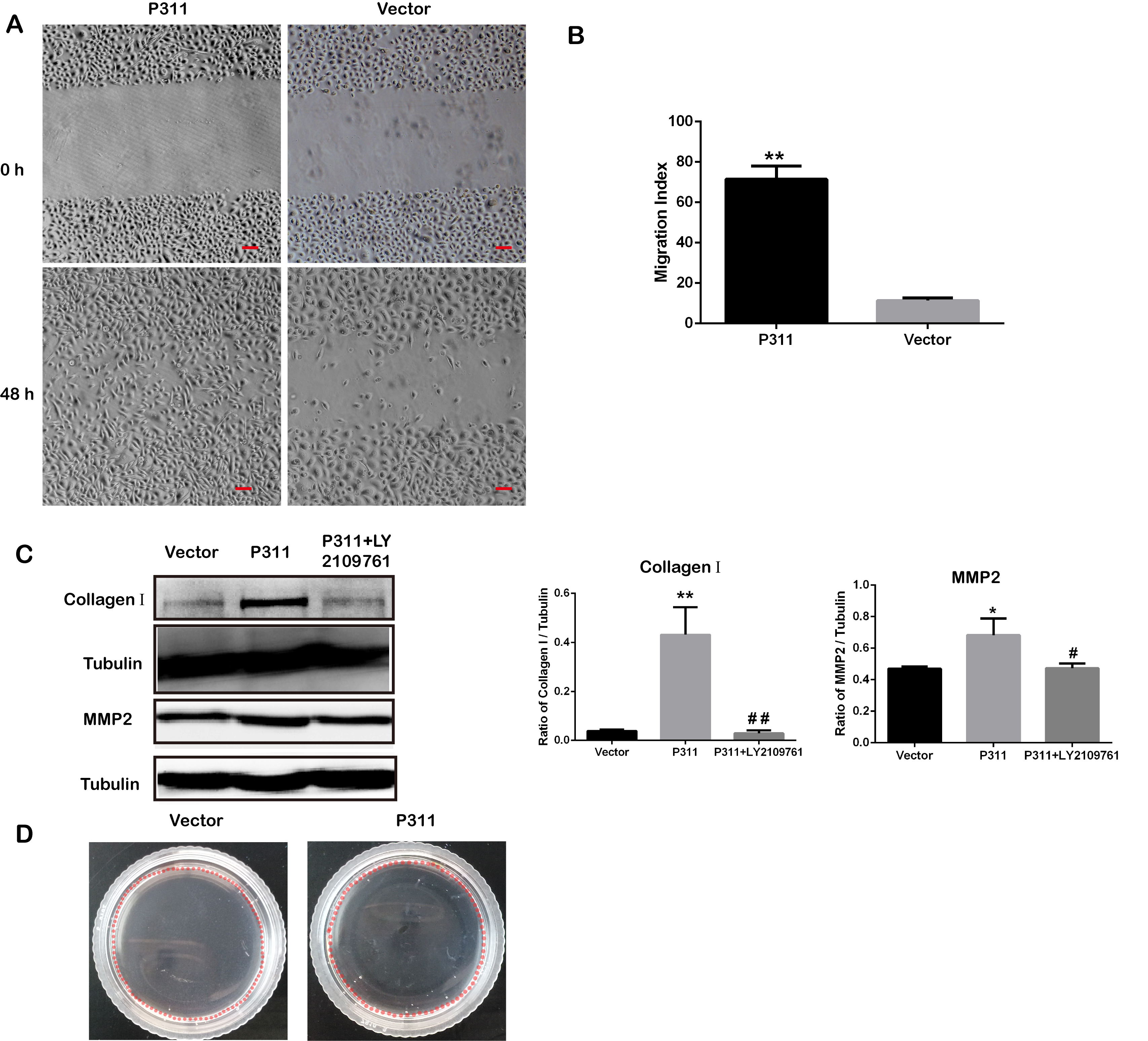
**

**Figure S5. The effect of P311 on the mesenchymal function of EpSCs.**

**A.** The *in vitro* analysis of EpSC motility was analyzed using a wound scratch model. Scale bar, 25 μm. **B.** The migration distance was measured after 48 hours and the migration index was calculated using the fraction of the migration distance in the original distance at 0 hour. **C.** Western blot analysis was performed to determine collagen I and MMP2 levels. ** P<0.01 for P311 vs. control vector; and ## P<0.01 for P311 vs. P311+LY2109761. **D.** The contraction of collagen was analyzed in the EpSCs. After the cells were transfected with the P311 or control vector adenovirus, they were cultured on collagen gels for one week. Red dotted lines indicated the gel border. The data represent the mean ± SEM, Student’s t-test.

**
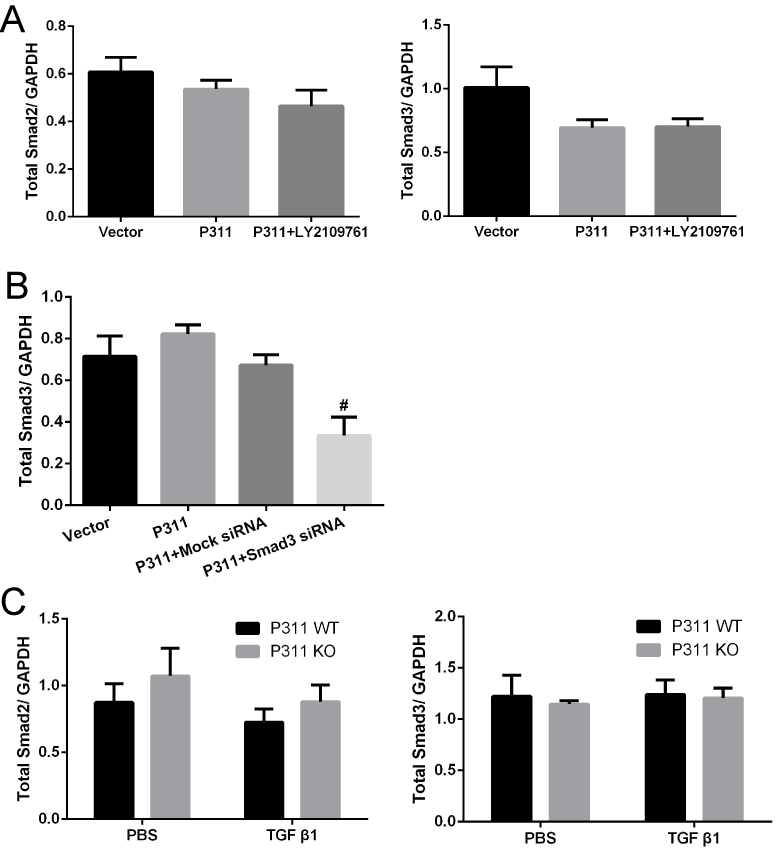
**

**Figure S6. The quantification of the total Smad2 and Smad3 protein.**

**A.** The total levels of Smad2 and Smad3 in primary EpSCs with or without LY2109761. **B.** The Smad3 levels after the stimulation of Smad3 siRNA. **C**. The total levels of Smad2 and Smad3 in P311 WT and KO EpSCs with or without exogenous TGFβ1. The data represent the mean ± SEM, One way ANOVA analysis.


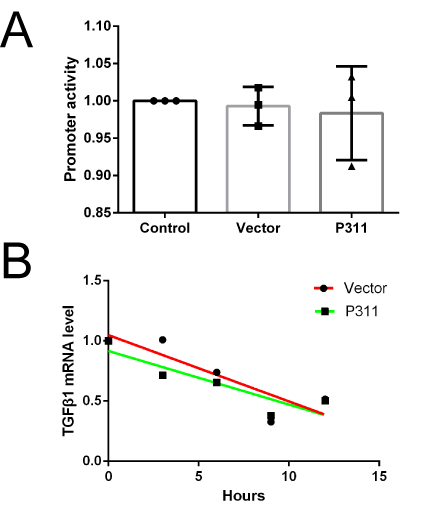


**Figure S7. The effect of P311 on TGFβ1 promoter activity**

Luciferase reporter analysis of TGFβ1 promoter activity in mouse EpSCs (n=3 per group). The data represent the mean ± SEM, Student’s T test.
